# Designs and Applications for the Multimodal Flexible Hybrid Epidermal Electronic Systems

**DOI:** 10.34133/research.0424

**Published:** 2024-08-09

**Authors:** Ding Li, Tianrui Cui, Zigan Xu, Shuoyan Xu, Zirui Dong, Luqi Tao, Houfang Liu, Yi Yang, Tian-Ling Ren

**Affiliations:** ^1^ School of Integrated Circuit, Tsinghua University, Beijing, China.; ^2^ Beijing National Research Center for Information Science and Technology (BNRist), Tsinghua University, Beijing, China.

## Abstract

Research on the flexible hybrid epidermal electronic system (FHEES) has attracted considerable attention due to its potential applications in human–machine interaction and healthcare. Through material and structural innovations, FHEES combines the advantages of traditional stiff electronic devices and flexible electronic technology, enabling it to be worn conformally on the skin while retaining complex system functionality. FHEESs use multimodal sensing to enhance the identification accuracy of the wearer’s motion modes, intentions, or health status, thus realizing more comprehensive physiological signal acquisition. However, the heterogeneous integration of soft and stiff components makes balancing comfort and performance in designing and implementing multimodal FHEESs challenging. Herein, multimodal FHEESs are first introduced in 2 types based on their different system structure: all-in-one and assembled, reflecting totally different heterogeneous integration strategies. Characteristics and the key design issues (such as interconnect design, interface strategy, substrate selection, etc.) of the 2 multimodal FHEESs are emphasized. Besides, the applications and advantages of the 2 multimodal FHEESs in recent research have been presented, with a focus on the control and medical fields. Finally, the prospects and challenges of the multimodal FHEES are discussed.

## Introduction

The flexible hybrid epidermal electronic system (FHEES) consists of both traditional stiff electronic components and flexible electronic components. FHEES is regarded as an intermediate state for flexible electronics in the development to intrinsically flexible systems given that the exploration of flexible integrated circuits (ICs) is still in its infancy and cannot be applied [1,2]. Although a plastic 32-bit ARM (a reduced instruction set computing [RISC] architecture)microprocessor based on indium–gallium–zinc oxide (IGZO) thin-film transistor technology has been presented by ARM Ltd. and PragmatIC Semiconductor Ltd., it is still a long way from achieving the same performance, density, and power efficiency as silicon-based ICs [3]. Inorganic semiconductor materials cannot withstand the large strain, and organic semiconductor materials have relatively poor carrier properties; thus, stretchable ICs are still not commercially available [4,5]. Even so, by partially utilizing flexible electronic components, the FHEES has successfully expanded the application scenario of electronic equipment. Especially in the flourishing Internet of Things, FHEESs can further release the ubiquitous information collection and analysis capabilities of artificial intelligent devices. For example, sweat analysis and drug delivery using soft bioelectronics on human skin provide a new route for noninvasive glucose level monitoring and management [6].

In numerous scenarios of human–machine interaction and healthcare, comprehensive physiological and environmental information is necessary to improve identification accuracy for the wearer’s motion modes, intentions, health status, etc. For human–machine interaction, whether to monitor human motion or simulate perception, hardware systems that can collect/generate multiple signals serve as the foundation for more sophisticated applications. For instance, researchers have demonstrated that combining physiological electrical signals and limb mechanical motion signals can realize more precise 3-dimensional (3D) control [7]. In the field of healthcare, a dual-channel graphene-based electromyographic and mechanical sensor flexible patch was designed to achieve speech recognition for patients with dysphasia in a noisy environment, which indicates that multimodal FHEESs is the future development trend for more accurate applications [8]. It is worth noting that, limited by the topic and length, this article mainly introduces the human–machine interaction and healthcare applications of FHEESs, while flexible electronics also promise an important role in environmental monitoring [9,10], safety protection [11,12], and other application scenarios [13,14]. If interested, detailed introductions can be found in these literatures.

Multimodal FHEESs can be classified based on different standards. Although multimodal FHEESs can collect multiple physiological or environmental signals, there is generally one main signal and other auxiliary signals. Accordingly, multimodal FHEESs can be divided according to the main collected signal, such as “electrochemical+” multimodal FHEESs, “mechanical strain+” multimodal FHEESs, “electrophysiology+” multimodal FHEESs, etc. This classification can be further refined into “sweat glucose+”, “throat vibration+”, etc. Common signal types and their response modes are summarized in Table 1. Another classification is concerned with the number of sensor types: integration of multiple sensors into a system or detection of multiple stimuli with a single sensor [15,16].

**Table 1.** Common signal types, response modes, and methods to avoid interference among different signals

Signal types			Response mode	Methods to avoid interference among different signals
Biophysical	Mechanical	Strain	Potential	a. Different sensing units
		Pressure	Resistive	b. Different sensing modes
		Acceleration	Capacitive	c. Isolation
		Vascular dynamics	Piezoelectric	d. Output discrimination
	Electrical	ECG	Biopotential acquisition from electrode–skin interface	a. Specific electrode position
		EEG		b. Signal filtering (software/hardware)
		EMG		c. Common mode feedback (avoid power line interference)
		EOG		
	Temperature		Resistive	a. Strain isolation
			Semiconducting	b. Strain calibration
			Optical	
Biochemical	Metabolites	Glucose	Electrochemical	a. Specific receptors
		Lactate	Optical	b. Microfluid handling
		Alcohol	Piezoelectric	c. Purification
	Electrolytes	Sodium	(relying on receptors, such as enzymes, antibodies, DNA, and whole cell)	d. Temperature calibration
		Ammonium		e. Strain isolation
		pH		
	Others	Heavy metal		
		caffeine		

**Fig. 1.**
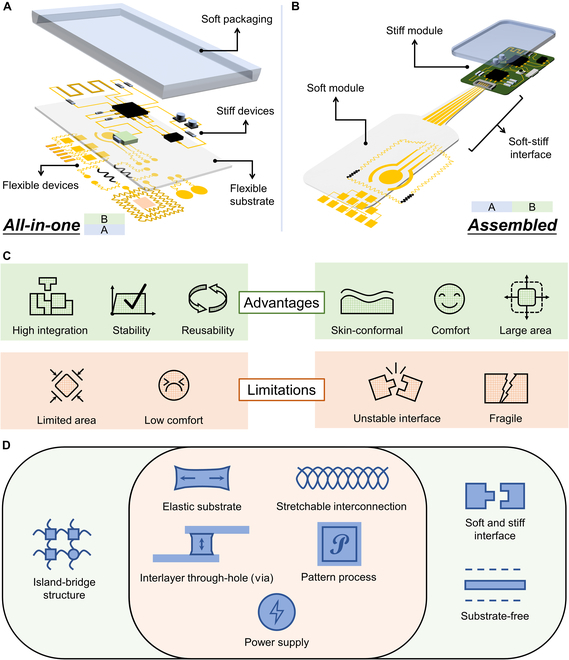
Based on different design strategies of the system structure, multimodal FHEESs are divided into 2 types: (A) All-in-one: adopting a stacked soft and stiff integrated structure, which generally consists of flexible components, flexible substrates, stiff components, and soft packaging. (B) Assembled: using heterogeneous (soft and stiff modules) separation integration technology to avoid direct contact between flexible and stiff components, and the interface between soft and stiff modules is the key component. (C) Advantages and limitations of all-in-one FHEESs and assembled FHEESs. (D) Unique and shared components of all-in-one FHEESs and assembled FHEESs.

However, the main challenge of the multimodal FHEES lies in how to integrate stiff components and flexible components, but the above classification method cannot highlight this challenge. For example, to stably fix stiff components in the system, adopting an ultra-thin substrate is impossible in many cases. Another example is that the placement density of stiff components should be reduced to leave enough space for flexible interconnection to ensure the overall flexibility of the system. These trivial and complex trade-off issues exist in the design process of every FHEES, but there have been no targeted reports to systematically organize these issues and corresponding solutions. Numerous excellent reviews have been presented based on applications or materials [1,17–21], and there are many sensor-oriented reviews [16,22–25], but specialized reviews for FHEESs classified by system structure remain absent. Here, according to different design strategies of the system structure, multimodal FHEESs are first introduced in 2 types: all-in-one and assembled. All-in-one multimodal FHEESs adopt a stacked soft and stiff integrated structure, which is generally composed of flexible components near the skin, flexible substrates in the middle layer, stiff components in the top layer, and soft packaging (Fig. 1A). All-in-one multimodal FHEESs typically exhibit a high degree of integration density, reusability, and functional stability at the cost of sensing area and wearing comfort, which is well-suited for the acquisition of small-area skin signals [26–28]. For assembled multimodal FHEESs, heterogeneous (soft and stiff modules) separation integration technology is used to avoid direct contact between flexible and stiff components (Fig. 1B). Assembled multimodal FHEESs can utilize thinner and larger flexible epidermal electronic components to achieve higher quality and more diverse skin-conformal acquisition, but the stability of the signal interface between the soft and stiff modules becomes crucial [29–32]. Besides, sensitivity to interference caused by fragile components is a challenging issue for assembled multimodal FHEESs. The advantages/limitations for all-in-one FHEESs and assembled FHEESs are summarized in Fig. 1C. From the perspective of components, island-bridge structures are only used in all-in-one FHEESs, while soft/stiff interface components and substrate-free design only appear in assembled FHEESs. They also have shared components, such as stretchable interconnections, and elastic substrates (Fig. 1D). Therefore, the 2 multimodal FHEESs, all-in-one and assembled, have different commonly used design strategies and are suitable for different application scenarios. This paper reviews and summarizes the research on multimodal FHEESs based on this binary classification.

In this review, we first introduce the design strategy and application scope of the all-in-one multimodal FHEES. In the design strategy, island bridge structure, substrate selection, stretchable interconnect, interlayer through-hole, and power supply will be introduced in turn. Human–machine interaction and healthcare applications are introduced in the application scope section of all-in-one multimodal FHEESs. Subsequently, we highlight the design purpose and strategy of assembled multimodal FHEESs. The soft–stiff interface is the key issue to be discussed initially, and the selection of substrate, patterning process, and stretchable conductors will be introduced in sequence. For the assembled FHEESs, limb-end sensing, physiological electrical signal acquisition, and large-area array sensing are the 3 typical applications. Finally, key issues that remain to be addressed are listed, and future perspectives and opportunities are discussed.

## All-in-One Multimodal FHEESs

In the design and manufacture of IC chips and printed circuit boards, multi-layer stacking technology improves the system integration density. In living organisms, multiple layers of biological tissues with different functions form complex and complete functional organs. Therefore, in the development of multimodal FHEESs, researchers use the same way to increase the functional density and improve working stability. At present, all-in-one multimodal FHEESs (with the stacked soft and stiff integrated structure) are the most extensively studied and widely used flexible electronic systems, because they break the conventional perception of electronic devices as either stiff or metallic.

### Design strategy

#### Island-bridge structure

In FHEESs, stiff devices based on traditional materials, such as silicon, cannot withstand large strain. In contrast, flexible substrates or components can still work normally within a certain strain range. Therefore, the island-bridge structure is designed for all-in-one multimodal FHEESs to appropriately distribute the overall strain of the system between the stiff component (small strain) and the flexible component (most strain).

**Fig. 2.**
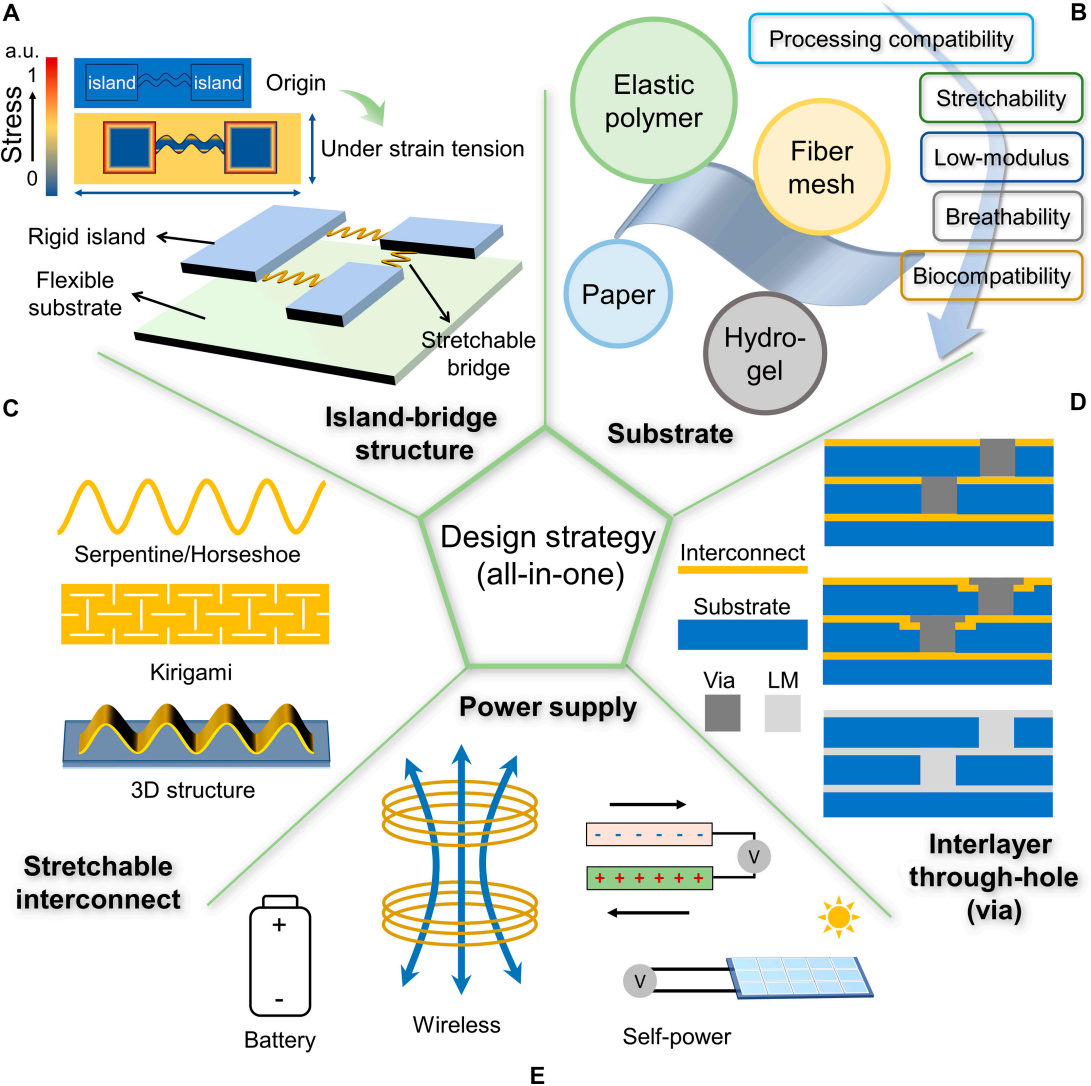
Design strategies for all-in-one multimodal FHEESs. (A) The illustrations of the island-bridge structure. The island-bridge structure can reasonably distribute the overall strain of the system. (B) The selection of substrates needs to consider processing compatibility, stretchability, low modulus, breathability, biocompatibility, etc. (C) Structural stretchable interconnects rely on 2-dimensional or 3D structures to achieve stretchability. (D) Multi-layer stacking is the main way to improve the integration. (E) Battery, wireless power, and self-power are 3 methods for power supply in all-in-one multimodal FHEESs.

In an all-in-one FHEES based on a basic island-bridge structure shown in Fig. 2A, rigid islands are embedded on a flexible substrate, and they are connected by stretchable bridges. In this structure, stiff components are placed on the strain-isolated rigid island, and the flexible part of the system absorbs almost all the strain. A typical work is a skin-inspired matrix network composed of 100 sensory island nodes connected by meandering bridge wires to imitate the multimodal sensory function of human skin [33]. Six types of sensors that are sensitive to strain were prepared on the island nodes through photolithography and sputtering processes. Aluminum films and polyamide (PI) meandering wires form the bridge for electrical conductivity, while poly(vinyl alcohol) or poly(dimethylsiloxane) are used as the substrate. Therefore, the presented systems have adjustable sensing range and large-area expandability characteristics.

In practice, in addition to the basic structure mentioned above, the design strategy of island-bridge structures has various forms. Firstly, the island can be embedded within the flexible substrate [34–36]. Researchers have used fluid as the substrate and then immersed the electronic system with the island-bridge structure into the fluid to construct an ultralow-modulus, highly stretchable FHEES with multiple physiological signal acquisition functions [34]. Second, since conventional IC chips can be regarded as rigid islands themselves, they can be directly interconnected by stretchable bridges to form the island-bridge structure [37,38]. In this form, the flexible interconnect is typically processed directly on the elastic substrate, and then stiff components are directly placed in the corresponding position and fixed, which simplifies the preparation process of the island-bridge structure. Third, the stress concentrated at the boundary of rigid islands and the flexible substrate can readily lead to mechanical and electrical failures of stretchable bridges or bonding points (illustration in the upper left corner of Fig. 2A) [39]; thus, using substrate mechanical engineering to build the island with mechanical gradients is widely studied [40]. Local reinforcement in the flexible substrates is used to avoid abrupt soft–stiff transition between rigid ICs and substrates, thus achieving greater stretchability [41]. Finally, in some works, although the island bridge structure no longer exists in its original form, the underlying idea of designing stress-isolated locations to protect fragile stiff components remains consistent. A typical example is that researchers directly embedded inorganic transistors in PI serpentine wires to overcome the trade-off between device density and stretchability of island-bridge structures. The middle part of the PI cladding between the top and bottom PIs is the neutral plane where the internal stress goes to zero when the PI cladding is flexed. Thin-film transistors are placed in sequence near the neutral plane of the serpentine cladding, thereby experiencing much less stress when the serpentine strings are subjected to stretching and twisting [5].

#### Substrate selection

In the initial development of FHEES, the substrate was generally composed of polymer materials [42,43]. In recent years, multimodal FHEESs on fiber substrates, paper substrates, and other substrates have also developed rapidly (Fig. 2B) [44–46].

The polymer substrate offers several advantages. Non-stretchable polymer substrates are compatible with traditional microelectronic processes, such as photolithography, metal deposition, ion etching, etc. For example, PI can be used as a stretchable flexible substrate through appropriate geometric structures, and it is resistant to chemical corrosion and high temperatures. The earliest epidermal electronic systems used PI substrates with curved structures to achieve effective elastic moduli, bending stiffness, areal mass density, and thickness that fit the epidermis. The epidermis electronic device incorporates many functional components such as electrophysiological electrodes, temperature sensors, strain sensors, light-emitting diodes, and photodetectors [47]. At present, all-in-one FHEESs based on the non-stretchable substrate are still the mainstream [33,48–50]. Polydimethylsiloxane (PDMS) and Ecoflex are the representatives of intrinsically elastic polymer substrates, which have similar Young’s modulus to human skin. For all-in-one FHEESs with higher requirements for stretchability and comfort, these materials are a superior choice to others [51–54]. To better understand the differences between polymer substrates, the 4 most important indicators are listed in Table 2: ultimate elongation, Young’s modulus, melting temperature, and solution resistance. The ultimate elongation is used to divide stretchable and non-stretchable substrates, and it determines whether a stretchable substrate meets the strain range requirements. Young’s modulus is the reflection of softness. At a specific thickness, substrates with lower Young’s modulus will have better skin conformality. Melting temperature and solution resistance affect the process methods in which they can be applied. In addition, 2 points need to be noted: (a) The characteristics of these substrates can be adjusted through structure, as mentioned above, PI substrates can realize stretchability through the serpentine structure; (b) other substrate characteristics such as thermal property, water adsorption, minimum processing thickness, and width can be explored in relevant reviews [55,56].

**Table 2.** Properties of different polymer substrates

Substrate	Ultimate elongation (%)	Young’s modulus	Melting temperature (°C)	Solvent resistance
PI	–	3–4 GPa	250–452	Good
PET	–	1–4 GPa	115–258	Good
PDMS	>100	0.5–2 MPa	–	Poor
PU	>300	0.1–10 GPa	180	Poor
Ecoflex	>150	20–125 kPa	–	Poor
SEBS	>50	~1 MPa	190–260	Good
PMMA	–	2–3 GPa	165–200	Good
Hydrogel	>1,000	10–1,000 kPa	–	Poor

Breathability is a challenging issue for all-in-one FHEESs. For systems with elastic polymer substrates, the holey structure is commonly adopted to improve air permeability [57]. However, fiber substrates naturally have extremely high porosity, which endows all-in-one FHEESs with brilliant breathability [58,59]. A monolithically integrated in-textile wristband integrates IC chips, sensors, a battery, and passive components on a permeable polyethylene terephthalate (PET) cloth. Such an in-textile all-in-one FHEES exhibits an excellent air permeability of 79 mm s^−1^ and a moisture permeability of 270 g m^−2^ day^−1^, which trumps commercial textile fabrics and shows more than one order of magnitude higher than commercial medical tapes and elastomers [27].

Additionally, there are some non-traditional substrates to meet special requirements. Here, paper-based substrates and fluid substrates are taken as representatives [60]. Paper-based substrates possess the advantages of low cost, flexibility, portability, hydrophilicity, eco-friendliness, and ease of handling [61]. A conductive hydrogel-paper patch was integrated with a flexible printed circuit board (FPCB) to simultaneously sense chemical–electrophysiological signals. Although they are all-in-one devices, the paper-based multimodal sensor (integrated with electrocardiogram [ECG] electrodes and glucose sensors) is disposable [62]. To further unleash the stretchable potential of the island bridge structure, silicone oligomer (Sylgard 184, without curing agent) was used as the fluid substrate. Following the bonding of the elastomeric superstrates, the silicone oligomer was subsequently injected by a needle to act as the highly stretchable substrate [34].

#### Stretchable interconnect

Stretchable interconnects are the decisive factor in the flexibility of the all-in-one FHEES. The connection of these interconnects to both flexible and rigid devices requires careful consideration, rendering the design of the stretchable interconnects in all-in-one FHEESs more challenging than in assembled FHEESs. For FHEESs, stretchable interconnects can be realized in 2 ways: structural stretchable interconnects and intrinsically elastic conductive interconnects. Structural stretchable interconnects play a dominant role due to their more stable conduction performance under strain conditions and compatibility with commonly adopted conductive materials. The design strategy of structurally stretchable interconnects in multimodal FHEESs is introduced here, and the intrinsically elastic conductive interconnect is described as the stretchable conductor in assembled FHEESs.

Generally, FHEESs use curved planar wires to realize stretchable interconnect (Fig. 2C). The curved design of the geometric structure endows rigid metal materials (e.g., gold, copper, aluminum, etc.) with certain stretchability. Representative stretchable interconnects include serpentine interconnects, horseshoe interconnects, and Kirigami structure interconnects [37,63]. When the curved planar wire is stretched, the strain is primarily concentrated in the bending region and the edge with the large curvature, but the conductive path located in other areas of the wire remains unaffected. Nevertheless, under high tensile strains (>10%), curved planar wires will still undergo complete fracture from the edge after repeated stretching [64,65]. To further improve the stretchability of planar curved wires, an effective method is to twist planar wires out of the plane when stretched. Therefore, the solid encapsulation was removed from the all-in-one FHEES. The FHEES used for display includes 3-axis accelerometer/gyroscope sensors, temperature sensors, and humidity sensors. It achieves a 3-layer, high-density, stacked integrated structure with stretchability of up to 20% [26].

Another effective method to improve the stretchable interconnect performance is to convert planar bent wires into 3D curved wires (Fig. 2C). As demonstrated by the example of helical wires, this approach can not only achieve higher stretchability but also disperse the stress induced by system deformation. Research has shown that the skin conformality of FHEESs based on helical wires can be improved [66]. Although the preparation process of 3D curved wires is more complex, researchers have developed a method of forming self-assembled 3D wires through pre-stretched elastic substrates. Firstly, the elastic substrate is pre-stretched, and then patterned planar wires are formed on its surface. The specific positions of the planar wires are fixed on the elastic substrate, and then the prestress of the elastic substrate is released to restore its original length. In this manner, the distance between the fixed positions of the planar wire is shortened as the elastic substrate recovers, and the non-fixed portion of the wires is twisted outward to form the 3D curved wires [51]. In addition to interconnecting wires, more complex 3D structures can also be obtained and applied in a wider range of fields through this approach [67,68].

#### Interlayer through-hole (via)

FHEESs on the plane need to leave sufficient space for stretchable interconnects and flexible devices to ensure the stretchability of the system. The most effective method of improving the integration of all-in-one multimodal FHEESs is through multi-layer stacking (Fig. 2D), which involves the design and implementation of interlayer through-holes (also known as vias). The interlayer through-hole should be designed to avoid fracture failure caused by the stress between layers during system deformation. However, the current interlayer through-hole is still mostly made of rigid metal materials, and the risk of fracture failure can only be reduced through structural design. Therefore, the stretchability of the all-in-one multimodal FHEES based on multi-layer stacking technology cannot be easily improved. The following section describes the interlayer through-hole based on traditional metal materials and new materials, respectively.

For all-in-one FHEESs with traditional metal materials for stretchable interconnects, their interlayer through-holes also use commonly adopted conductive materials to avoid heterogeneous integration [26,28]. Opening holes and then soldering is the most common way. In an all-in-one stretchable electronic system with 4 layers, selective laser ablation is used to remove the silicone substrate. Through-holes (open on both sides), buried holes (open on neither side), and blind holes (open only on one side of the stretchable system) can be realized by this method. Then, solder paste is dispensed into the holes via screen printing [28]. The screen printing method was later changed to a simpler way of filling silver pillars (In_97_Ag_3_) into vertical holes [26]. Finally, bonding is completed by heating reflow soldering. The through-hole achieved by this method still largely depends upon traditional materials and does not have strain capacity; thus, new materials-based through-hole preparation methods have been explored.

Conductive flexible composite materials and liquid metal (LM) are 2 options for new through-holes. Interlayer through-holes formed by conductive flexible composite materials are suitable for the majority of scenarios [37,69]. A single droplet printing-based method was proposed to create core–shell vertical interconnect access using composite materials. First, the nickel (Ni) microparticles/PDMS/silicone resin mixture ink was embedded inside the PDMS substrate, and then ferromagnetic Ni microparticles were self-assembled into the center of the mixture by a highly focused magnetic field. This vertical alignment of Ni particles offered a high degree of electrical conductivity in the vertical direction. The core–shell structure simultaneously satisfies both electrical and mechanical requirements for a stretchable interlayer through-hole [70]. LM-based through-holes are mostly used for all-in-one FHEESs with LM circuits. As a representative, the 3D flexible electronics system developed by Li et al. has garnered specific attention. They utilize the solid–liquid phase transition and plastic deformation characteristics of the gallium alloy to build 3D hybrid circuits. At low temperatures, gallium alloy wires retain their deformable solid state and can be shaped into circuits. These shaped alloy wires and rigid electronic devices are encapsulated in an elastomer before being heated above their melting temperature. This process effectively solves the leakage problem of the LM and eliminates the additional process steps in the formation of interlayer through-holes [71].

#### Power supply

The 3 principal energy supply solutions for all-in-one FHEESs are battery, wireless power transfer, and self-power supply (Fig. 2E).

Battery-based power supply is a traditional and common method, but rigid batteries not only increase system weight and occupy available space, but also introduce stress concentration issues at the soft and rigid interfaces in all-in-one FHEESs, reducing the comfort and reliability of the system [26,28,72]. In battery-powered FHEESs, the battery is generally placed at the top layer of the system while minimizing system power consumption to ensure that a smaller battery can provide the necessary power for system operation [62,73]. There are some power supply solutions with flexible printing batteries, but the energy they can provide is still insufficient to meet the long-term operational requirements of a fully functional all-in-one multimodal FHEES. The trade-off between the mechanical and electrochemical properties of stretchable batteries remains unresolved [74,75].

Wireless power transfer (WPT) includes near-field transfer techniques and far-field radio-frequency techniques. Due to the directional transmission and low transmission efficiency, it is difficult to apply the far-field power transfer technique (radio-frequency transmission and reception) in FHEESs at present [76]. Near-field transmission depends on inductive coupling and magnetic resonance. It has high efficiency of near-field communication (NFC), but the transmission distance is limited. A multimodal wireless epidermal electronic system powered by NFC was designed for existing vital signs monitoring in the neonatal intensive care unit, which demonstrates the potential application of WPT in applying power to epidermal electronic systems in a small space [38]. To make FHEESs more flexible in a variety of scenarios, battery and WPT power hybrid supply schemes have also been reported [49]. With the continuous improvement of the energy transfer efficiency of WPT, it will be more widely used in all-in-one FHEESs to reduce system weight.

Self-powering supply is a potential way. There are already many self-charging flexible devices, such as photoelectric flexible devices, triboelectric nanogenerators (TENGs), bioelectrochemical flexible devices, and so on. However, the biggest challenge for ambient energy harvesters is to produce enough power for an entire sensing system. Beyond triboelectric and photovoltaic energy conversion, the power generation efficiency and/or power density of most current technologies is insufficient to support complex sensing systems. TENGs can convert the mechanical energy of motion into electrical energy via coupling of inductive and triboelectric effects, allowing them to be used when FHEESs are far away from the sunlight or wireless power transmitters [77,78]. Song et al. presented a wireless battery-free wearable sweat sensing system powered by the FPCB-based TENG (FTENG). To collect the mechanical energy caused by a sliding motion between the side of the torso and the inner arm more effectively, the stator of FTENG was fixed on the side torso, and the slider of FTENG was attached to the inner part of the arm. The self-powered multimodal system accurately measured the pH and Na^+^ levels of sweat 5 times during the wearer’s 30-min exercise [79]. Compared with TENG, the energy conversion efficiency of photoelectric devices is higher. Therefore, self-powered FHEES implemented with a perovskite solar cell can support more diverse signal acquisition and longer working time [80]. At present, FHEESs with self-power supply still need the cooperation of batteries to achieve 24-h operation.

### Application scope

All-in-one multimodal FHEESs pursue integration and stability. Their coverage of the human epidermis is limited, and the various physiological signals that can be collected are generally located at the same position in the skin. This determines that the signal combination of all-in-one multimodal FHEESs can be divided into 2 situations: (a) there is no strict requirement for the measurement position for all the signals collected by all-in-one multimodal FHEESs; (b) the collection of a certain type of signal has a clear measurement position while other signals do not. The majority of all-in-one multimodal FHEESs conform to the second. That is, the placement of all-in-one multimodal FHEESs on the skin is generally determined by a major signal, which also determines the application fields of the all-in-one multimodal FHEES.

#### Human–machine interaction

In the Internet of Things era, massive data are collected and processed, and the ultimate service object of this information is people. Compared with the speed of data exchange between machines, the efficiency of people in obtaining and outputting information from the outside world is very low. Therefore, the existing human–machine interaction mode must be continuously improved to better receive, use, and schedule massive information. Multimodal FHEESs may subvert the existing human–machine interaction technologies, bringing a more natural and comfortable mode. Human–machine interaction can be conceptualized as an interface tissue, where information flow is divided into human-to-machine and machine-to-human.

**Fig. 3.**
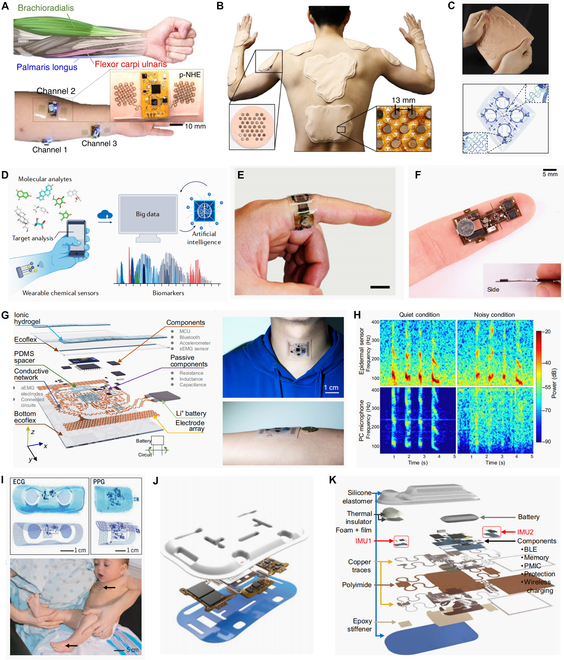
Applications for all-in-one multimodal FHEESs. (A) FHEESs are placed on the arm to collect the multi-channel EMG from the muscles under the skin. Reprinted/adapted from Kwon et al [81]. (B)(C) Al-in-one FHEESs for virtual or augmented reality. Copyright 2019, Springer Nature, reproduced with permission [49,50]. (D) Wearable chemical FHEESs can be used for biomarker discovery. Copyright 2022, Springer Nature, reproduced with permission [86]. (E) All-in-one multimodal FHEESs for non-invasive female hormone monitoring. Copyright 2023, Springer Nature, reproduced with permission [73]. (F) Researchers integrated a sweat PDMS microchannel, a thermal actuator, and two thermistors into a skin-interfaced, wireless all-in-one FHEES. Copyright 2021, Springer Nature, reproduced with permission [52]. (G) The structure and images of an all-in-one multimodal FHEES for throat monitoring. Reprinted/adapted from Xu et al [72]. (H) All-in-one FHEESs for throat monitoring perform better than the PC microphone under noise conditions Copyright 2016, AAAS, reproduced with permission [53]. (I) Binodal, wireless FHEESs for neonatal intensive care. Reprinted/adapted from Chung et al [38]. (J) All-in-one multimodal FHEESs with holey architectures for wireless physiological monitoring. Copyright 2021, Wiley, reproduced with permission [57]. (K) The structure illustrates a differential cardiopulmonary monitoring system for artifact-canceled physiological tracking. Copyright 2021, AAAS, reproduced with permission [89].

The transmission of information from human to machine requires accurate recognition of human intentions. It is an important way to recognize human motion patterns using limb movement. Limb movement signals (such as angle, acceleration, and skin strain) can be collected in a limited skin area to reflect the posture of the limb, which is suitable for all-in-one multimodal FHEESs [47,81]. The common position for these devices is the forearm. The acceleration and angle are used to reflect the twisting posture of the forearm, and the electromyogram (EMG) and the skin strain can reflect the force of the muscles; thus, the all-in-one multimodal FHEESs can be used to recognize the motion pattern of the arm accurately (Fig. 3A). For human-to-machine applications, the number of recognizable patterns and the recognition accuracy are 2 key parameters that determine the efficiency of the interaction process. Increasing the number of signal channels and adopting multiple types of sensors in all-in-one FHEESs can greatly improve them. For example, multiple printed EMG all-in-one devices can be placed on the forearm to monitor the motion of target muscles; thus, different finger motions are divided and achieve an accuracy of about 99% for 7 classes [81]. By wearing an 8-channel dual-mode (triboelectric and piezoelectric) gesture recognition device around the wrist, researchers can achieve a maximum accuracy of 92.6% in recognizing 26 letters [82].

Mechanical strain/pressure, electrophysiology, acceleration, and angle are the 4 main signal types used for human-to-machine interaction. For mechanical strain/pressure, flexible strain/pressure sensors are placed on the bottom of the all-in-one FHEES to deform together with the skin, causing changes in resistance, capacitance, or inductance, or reflecting the deformation process through active signals such as piezoelectric and triboelectric electricity [9]. The electroencephalogram (EEG) and EMG are commonly used electrophysiological signals. EEG is generated with the intention of the human brain, reflecting the brain’s neural activity. The stretching and contraction of muscle cells are accompanied by changes in cell potential, which EMG is born from. All-in-one FHEESs collect these weak electrophysiological signals from the skin by flexible electrodes, followed by differential amplification, filtering, analog-to-digital conversion, and classification recognition. Acceleration and angle are commonly acquired by a MEMS chip, which includes a MEMS accelerometer and a MEMS gyroscope. The core of MEMS accelerometers lies in their micromechanical structure, which typically includes a movable “oscillating mass block” and fixed electrodes. When the chip is subjected to acceleration, the mass block will experience displacement due to the inertia force, which will change the distance between the mass block and the fixed electrodes, thereby causing a change in the capacitance value. The acceleration magnitude of the object can be derived by measuring the change in capacitance value. As for the MEMS gyroscope, it generally consists of a “rotating mass block” and a supporting structure. When external rotation acts on the gyroscope, the rotating mass block produces a gyroscopic effect, causing it to tilt or rotate. The supporting structure measures and controls torque by piezoelectric or inductive effects. Electrophysiological acquisition is sensitive to the electrode–skin interface. Even if the skin under stress/pressure is not located at the electrode, changes in the electrode–skin interface caused by skin deformation can still cause marked changes in the baseline of physiological electrical signals [44]. When motion is not intense, low-frequency baseline disturbances (<5 Hz) can be removed through data filtering, but disturbances within 10 to 1 kHz will seriously affect the readability of physiological electrical signals. To minimize this interference, electrodes should be placed far from the strain/pressure sensors, and try not to place stiff components above the electrodes. Besides, strain and pressure stimuli are always coupled with each other for flexible sensors. Therefore, a decoupled signal output is necessary if a single sensor is used to measure both strain and pressure. If the strain and pressure sensors are separated, the separated pressure sensor should include the strain isolation structure to avoid strain interference, and the location of the strain sensor should also avoid pressure stimulation [16].

Information feedback from machine to human is a key step in the formation of a complete human–machine interaction cycle, and all-in-one FHEESs promise to achieve novel feedback devices. The researcher combines specially designed micro-mechanical actuators, flexible substrates, flexible interconnects, and control circuits in a stack-integrated manner to form an all-in-one FHEES for virtual or augmented reality (Fig. 3B and C) [49,50]. The device can receive multimodal stimuli like real human skin and give skin the corresponding feedback feeling. Its application can be extended to social media devices, prosthetics, and so on.

Mechanical deformation and electrotactile are always used to simulate the tactile feedback. Mechanical actuators convert electrical signals into mechanical deformation and skin receptors will translate this stimulation into tactile nerve signals. The electrotactile method directly inputs current into the skin and stimulates skin receptors to induce virtual tactile sensing. A study has presented that the combination of mechanical actuators and the electrotactile can comprehensively stimulate tactile receptors at different depths in the skin to stimulate the tactile experience in different frequency bands. Besides, temperature feedback is commonly based on heating resistance wires, which convert electrical energy into heat energy to change the skin temperature [83]. The density and spatial distribution of feedback units and the feedback modes determine the complexity of interface functionality. Grounded haptic devices could offer the feedback of weight from their controlled machine, but its feedback only applies to the low-frequency mechanics of the entire palm and 5 fingers [84], while joysticks usually provide mechanical vibration or temperature from a single actuator [85]. Compared with them, the all-in-one multimodal FHEES based on flexible interconnect and electrodes promises a more precise and lightweight strategy that has achieved the integration of 3 modes with 15 feedback pixels in a single palm device [83].

#### Health monitoring

*Body fluid monitoring.* Inorganic ions in body fluids can reflect the acid–base balance of the human body. Small-molecule substances in body fluids, such as glucose and vitamins, are important for monitoring the real-time state of the human body, while large molecules, such as proteins and nucleic acids, are key biomarkers for many diseases. With the advent of an aging population and the concomitant increase in public awareness of health issues, the screening of potential biomarkers through alternative body fluids, such as sweat, saliva, tears, and interstitial fluid (ISF), represents a non-invasive and highly promising application direction (Fig. 3D) [86,87]. FHEESs for body fluid monitoring can be placed on any skin that does not affect the user’s daily life. In addition, they contain integrated epidermal sensors that enable rapid, sensitive, and selective detection of a broad range of targets in alternative body fluids and analysis circuits for signal preprocessing and wireless data transmission. Therefore, the all-in-one multimodal FHEES with high integration, small area, and stacked structure is the optimal choice.

In body fluid monitoring, in addition to the monitoring of the main target biomarkers, some other physiological signals for calibration and comprehensive identification are acquired. Ye et al. [73] developed an all-in-one multimodal FHEES worn on the index finger for non-invasive female hormone monitoring (Fig. 3E). The system contains an autonomous sweat induction module (iontophoresis), a potentiometric pH sensor, a resistive skin temperature sensor, and an impedance ion strength sensor for real-time sensor calibration. Its electronic circuits for signal processing and wireless communication are placed on the top layer. This miniaturized system has mechanical flexibility, enabling personalized female hormone monitoring and analysis during menstrual cycles.

In another case, the integration of multiple sensors and actuators in an all-in-one multimodal FHEES is to obtain a key biometric indicator. For example, it is valuable to monitor the flow rate, cumulative loss, and temperature of sweat for the diagnosis of thermoregulatory disorders and illnesses related to heat stress. However, obtaining accurate, continuous estimates of these parameters through one sensor is challenging. Kwon et al. integrated a sweat microchannel, a thermal actuator, and 2 thermistors into a skin-interfaced, wireless system. The incorporation of active heating devices reduces the sensitivity to environmental fluctuations, and 2 thermistors cooperate to measure the temperature difference and distinguish the flow rate of sweat in the microchannel [52]. Besides, by adding more microfluidics structures, the proposed system can be modified to enable colorimetric detection of sweat pH, and the concentrations of chloride, glucose, and creatinine (Fig. 3F).

For body fluid monitoring, detection techniques can be classified by electrochemical or optical detection. Electrochemical sensors, including amperometric sensors, potentiometric sensors, and voltammetric sensors, can convert target analyte information into a measurable electrical signal, which usually immobilizes bioreceptor molecules on the surface of the electrode to interact with the target analyte. Optical sensors rely on modifying light absorbance or emission regulated by chemical or biological reactions. For example, the electron state of chromophore molecules can be changed by external stimuli, which leads to the absorption of different wavelengths, i.e., color changes. For body fluid sensors, the measurable concentration range of the target measurement substance is a key indicator, but readers should pay attention to the stable performance of the system. Various parameters (such as temperature, pH, and reactive oxygen species levels) will affect the accuracy of the system. For fluid extraction and sampling, options include microneedle, reverse iontophoresis, and microfluidic/microchannel. Microneedle-based detection is a minimally invasive method used to make contact with the body fluid directly. Reverse iontophoresis allows the extraction of the ions and neutral molecules from the ISF to the skin surface. Microfluidic/microchannel methods need sweat-stimulating electrodes to stimulate perspiration, but they can realize in situ intermittent storage and controlled handling of the secreted sweat without dilution, mixing, or cross-contamination. To avoid interference among various signals, sensors with different bioreceptors should be separated in space, and microfluidic/microchannel methods can be used to handle or mix target fluid [86]. The influence of strain on body fluid monitoring applications has been less considered for many reports due to the small sensing area and non-stretchable substrate. If the sensor of body fluid analysis undergoes strain, the characteristics of the electrode and the contact between the electrode and the target fluid will both change, which will change the result curve, affecting the accuracy of the measurement. Bending will also lead to small strain at specific locations, but the impact of bending can be overcome by designing the sensor position and area appropriately [73].

*Throat monitoring.* Throat monitoring devices are urgently needed for patients with language disorders or those who have undergone throat surgery. Besides, throat monitoring is an effective way of achieving speech-based communication when sound signals are seriously disturbed by external noise. The muscular movements of the throat or the acoustic vibrations of the throat can be monitored by the surface of the throat skin. However, the large size and rigid structure of traditional devices present a challenge in developing electronic devices that can be worn on the throat for a long time, making the research on throat monitoring one of the application hotspots for all-in-one multimodal FHEESs.

Because the muscle motions and vocal organ vibrations contain recognizable speech characteristics, throat monitoring usually involves mechanical and EMG signals [8,88]. With Ecoflex elastic polymer material as the substrate and serpentine copper wire as the interconnect, the microcontroller (MCU), Bluetooth, and acceleration chips are integrated to form an all-in-one FHESS patch, which can be stretched up to 30% to meet the skin strain range. This throat monitoring patch wirelessly measures and analyzes various vibrations and muscle activity directly from the skin. In addition to the surface electromyographic (sEMG) signal, the triaxial accelerometer integrated into the patch can monitor strenuous physical movements (such as walking and jumping) and subtle physiological activities (such as heart rate and breathing). On the one hand, the measurement of these multimodal signals provides more physiological information about the wearer, and on the other hand, the recognition accuracy of the throat acoustic vibration signals in the motion state is further improved. Based on this throat multimodal FHEES, researchers used a feature extractor of the CNN algorithm to classify 13 general features in 14 healthy subjects and 2 patients (1 myasthenia gravis and 1 laryngeal cancer) with an accuracy of 98.2% (Fig. 3G) [72]. Besides health monitoring, the throat monitoring all-in-one multimodal FHEES serves as a natural human–machine interface. Liu et al. demonstrated the effective function of throat all-in-one FHEESs for language acquisition and recognition under noise (4 classes with 90% accuracy), with a length of only 2 cm and a width of no more than 1 cm (Fig. 3H). With the help of this throat patch FHEESs, users can complete computer game tasks naturally and smoothly through voice [53]. In all these works, mechanical sensors and EMG electrodes are placed on different layers to reduce area and interference. Although stiff encapsulated chips are almost unaffected by other signal interference, the crosstalk issue between flexible mechanical sensors and EMG electrodes remains to be explored. There are few systematic studies on the strain effects of throat skin deformation on the system, which may hinder the development of their practical applications.

*Other applications*. Other representative applications include single-lead ECG, heart rate, body temperature, pulse, and blood oxygen [48,54,89]. Some of these physiological signals have specific requirements for placement, such as the all-in-one multimodal FHEES containing single-lead ECG, which should be placed in the chest to acquire ECG. While the measurement of other physiological signals does not strictly require a specific collection location; thus, the placement position of the all-in-one FHEES gives priority to comfort.

ECG is one of the fundamental methods for accessing the condition of patients’ vital signs, but its collection requires specific electrode locations. With an all-in-one FHEES patch integrated with single-lead ECG electrodes and photoplethysmography sensors, Chung et al. successfully implemented wireless vital signs monitoring of neonates in the intensive care unit. The flexible patch with a wireless power supply and wireless data transmission eliminates the need for numerous wires and bulky electrodes of traditional monitoring equipment, which greatly improves the comfort of the monitored neonates. Additionally, despite its placement on the chest of neonates, this small and thin epidermal device does not interfere with the physical contact between parents and neonates and does not affect the medical imaging examinations (Fig. 3I) [38]. The reasonable pore design of the all-in-one multimodal FHEES facilitates the enhancement of breathability and stretchability (Fig. 3J) [57]. For performance parameters, a well-measured ECG signal can identify various characteristic wave groups for medical diagnosis and the quality evaluation of the ECG measurement can be achieved through intuitive comparison with measurement results from standard equipment or signal-to-noise ratio comparison.

For signal acquisition that does not require precise measurement position, the system’s placement is more in line with the user’s comfort needs. Jeong et al. [89] proposed a skin-interfaced device in which one of its inertial measurement units (IMUs) is located at the suprasternal notch (SN) and the other at the sternal manubrium (SM) (Fig. 3K). Due to the device being attached to a skin area far from frequent strain, the system can achieve real-time measurement of heart and respiratory rates under intense exercise (such as playing basketball and swimming). Besides, differential operation by 2 identical units at different locations to overcome the limitation of motion artifacts is an inspiring strategy for all-in-one multimodal FHEESs.

## Assembled Multimodal FHEESs

Although many FHEESs have been classified as all-in-one, they have developed characteristics toward assembled multimodal FHEESs. For example, the area of the sensor part is larger than the top circuit parts [38,48,53,54], or the flexible sensor part is no longer located directly below the top circuit parts [37,41,73,79]. The assembled multimodal FHEESs separate the soft module from the stiff module and then connect it through appropriate soft–stiff interfaces to form a complete functional system (Fig. 1B). From a usage perspective, the assembled structure can leverage the advantages of soft modules and stiff modules, respectively, achieving both comfortable and powerful FHEESs. From a production perspective, separating the preparation processes of soft and stiff modules can avoid the incompatibility between flexible and rigid components, thereby enhancing their stability performance, and reducing costs. The research on multimodal flexible sensor components predates systems, and flexible sensors are often connected to stiff measuring instruments in the laboratory for test. Therefore, researchers of multimodal flexible sensors unconsciously adopted the separated structure to finish their tests [44,45,90–93]. With the rapid development of epidermal electronic system research, the assembled multimodal FHEES is becoming a trend. Understanding the design strategy and application scenarios of assembled multimodal FHEESs will assist researchers in identifying research direction and making effective efforts.

### Design purpose and strategy

#### Soft–stiff interface

**Fig. 4.**
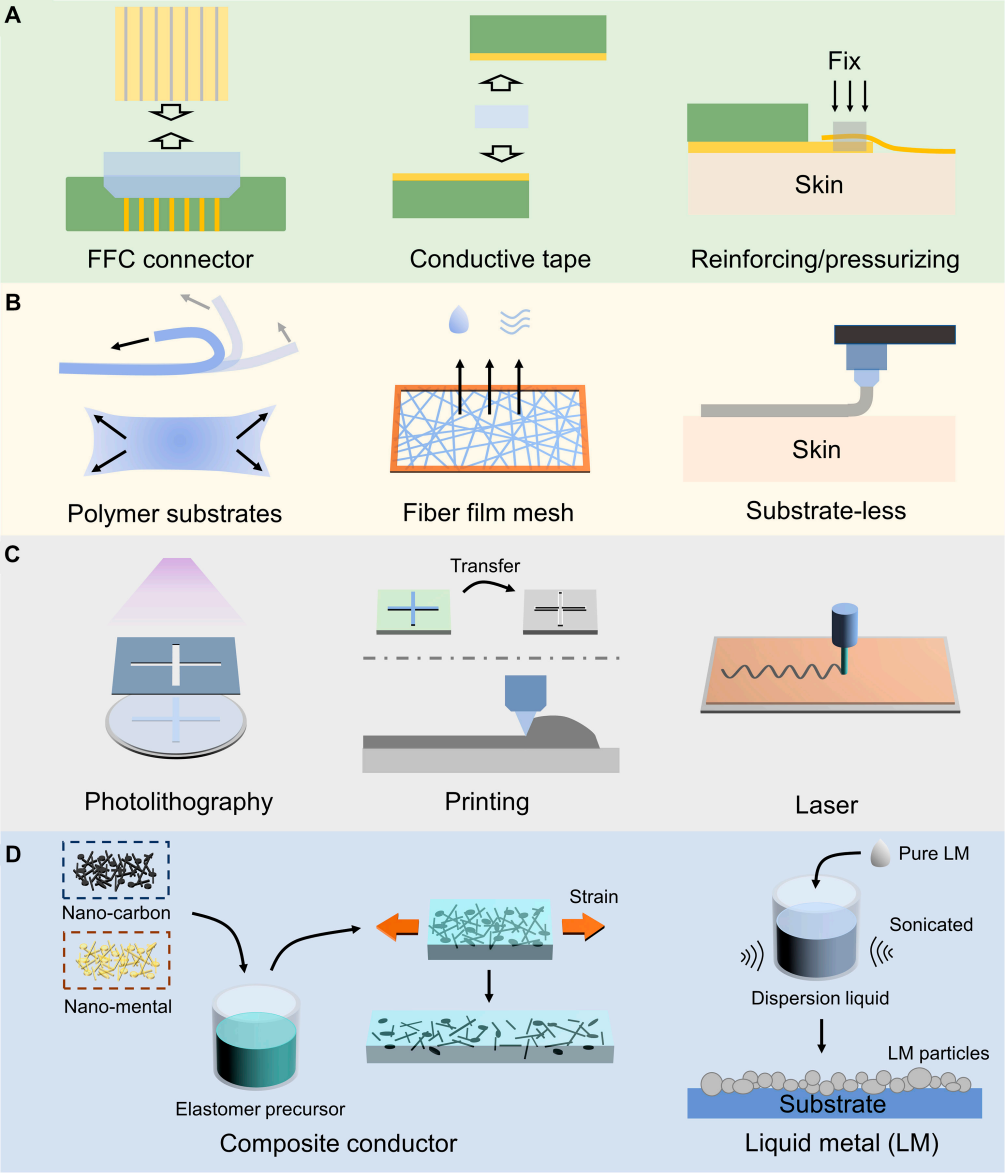
Design strategies for assembled multimodal FHEESs. (A) Implementation methods of soft–stiff interfaces for assembled multimodal FHEESs. (B) The substrate selection for the soft modules of assembled multimodal FHEESs. (C) Pattern processes for the soft modules of assembled multimodal FHEESs. (D) Stretchable conductors can be classified into composite conductor and liquid metal.

Unlike the widely existing soft–stiff contacts in the all-in-one FHEES, the assembled multimodal FHEES features a soft–stiff interface, which plays a critical role in facilitating communication between the soft and stiff modules. Given that the soft and stiff modules in assembled multimodal FHEESs are manufactured separately and exhibit high reliability, the overall stability of the system depends on the performance of the soft–stiff interface. In some reports on flexible devices, the soft–stiff interfaces used in the tests are still traditional methods, such as welding and silver paste. However, soft–stiff interfaces formed in these ways are fragile when subjected to strain and are therefore unsuitable for the construction of assembled multimodal FHEESs [94–98]. The following section will classify and introduce the emerging soft–stiff interfaces in the reported assembled multimodal FHEESs (Fig. 4A).

For multimodal flexible sensors made with non-stretchable flexible substrates, the standard flat flexible cable (FFC) connector can be used as the soft–stiff interface [32,83,99,100]. Song et al. present a universal semisolid extrusion-based 3D printing technology to fabricate multimodal electronic skin. The method allows for rapid customization of printing on flexible substrates to form sensors based on different materials. Researchers have demonstrated a soft module that integrates devices such as pH sensors, glucose sensors, temperature sensors, pressure sensors, etc., which has a standard spacing electrode interface on one end. Finally, it is connected to the wireless electronic module through an FFC connector [100]. For arrayed flexible sensors, FFC connectors with high-density connection points are widely used. Song et al. report a scalable approach to integrate microscale devices onto flexible polymer substrates with interconnected arrays. As an application demonstration, they prepared an 8×8 array patch containing 64 sensing units, with each sensing unit integrated with a pad for bioelectrical potential collection, as well as a transistor for signal amplification and a transistor for selective reading. The signal output of this array is through a ZIF connector (one of the connectors for FFC) containing 16 electrodes [101]. One of the limitations of the FFC connector is that it cannot be used for stretchable thin-film substrates.

The conductive tape is a method for the interconnection of soft and stiff modules in assembled FHEESs. This connection process does not involve high temperatures and pressures, thereby preventing damage to the substrate in the soft module. The anisotropic conductive film (ACF) is one of the representatives. It is a composite composed of fine conductive particles that are uniformly dispersed in the adhesive matrix. During the assembly process, the conductive particles approach each other in the compressed area to form electrical connections, while other areas maintain insulation characteristics, ensuring automatic pad alignment and connection [102]. Therefore, ACF can be used for the connection of scaling microelectrode arrays in high-density soft modules [103,104]. Besides, a plug-and-play flexible interface solution has also been developed. The self-adhesive styrene-ethylene-butylene-styrene (SEBS) was used as the substrate, and then gold (or silver) nanoparticles were deposited on the SEBS by a vacuum thermal evaporator. By regulating the concentration of metal nanoparticles distributed on the surface of SEBS, conductive interfaces with self-adhesive properties can be formed. The plug-and-play interface connects modules (soft–soft connections or soft–stiff connections) by simply pressing without the use of pastes and presents 600% and 180% mechanical and electrical stretchability [29].

Another way is to build soft–stiff interfaces by locally reinforcing or pressurizing to form close contact. This type of interface generally requires the extension of the soft module to a planar and relatively static position on the skin, such as the wrist, and then the implementation of reinforcement components to connect the stiff module [30]. An example is a graphene e-tattoo (GET) for unobstructed ambulatory electrodermal activity sensing on the palm. Because the measurement position is within the palm, the soft module adopts lightweight and transparent GETs. To connect the GETs with the stiff module, researchers used a serpentine Au/PI ribbon to extend the GETs to the wrist, and then the stiff module was pressed onto one end of the ribbon through an interlayer conductive rubber [105]. In this way, the soft module is arranged on the skin surface that undergoes significant and frequent strain. The stiff module, on the other hand, was placed in the comfortable wearing position.

#### Substrate selection

In assembled multimodal FHEESs, the flexible substrate is not responsible for supporting stiff components, allowing for thinner and larger designs. Figure 4B shows that the substrate selection is divided into 3 types: polymer substrate, fiber mesh substrate, and substrate-less.

PI and PET have good chemical inertness and high-temperature resistance (Table 2), which enable them to be compatible with various processing techniques, thus laying the foundation for the preparation of multiple sensors on the same substrate [6,73,106]. Assembling integrated wearable sensor arrays prepared on PET substrates with processing FPCB circuits can achieve mechanically flexible and fully integrated wristband FHEESs for in situ perspiration analysis [107]. Sweat metabolites (such as glucose and lactate) and electrolytes (such as sodium and potassium ions), as well as the skin temperature (to calibrate the response of the sensors), can be collected, pre-processed, transferred, analyzed, and sent within the proposed assembled multimodal FHEES. PDMS is another commonly used polymer material [33,108]. Due to its excellent mechanical properties, optical transparency, water resistance, and biological compatibility, PDMS has become the preferred substrate for microchannel devices. This determines that the use of PDMS is widespread in the implementation of assembled multimodal FHEESs for sweat electrochemical analysis [109,110].

Wearing imperceptibility is pursued by assembled multimodal FHEESs, and the ultra-thin fiber substrate prepared by electrospinning is a good choice in terms of both air permeability and stretchability [111]. A variety of efficient and low-cost ways to fabricate sensors on fiber substrates have been reported, such as screen printing, sputtering, filtration, and so on [58,112,113]. However, if sensors based on the fiber substrate adopt the all-in-one structure to build FHEESs, additional functional layers on the fiber substrate will destroy the air permeability and ultra-thin characteristics. Therefore, multimodal assembled FHEESs based on the fiber substrate attract considerable interest. Inspired by the active liquid transport phenomenon observed in nature, Xu et al. proposed an electronic skin composed of multi-layer fiber. The hydrophobic fiber layer was directly electrospun on the pre-electrospun hydrophilic fiber layer, which leads to directional liquid transportation. Therefore, the soft module based on fiber substrate performs ultrafast perspiration-wicking capability. The fiber-based flexible device minimizes the measurement error of skin hydration and temperature due to perspiration and reduces the noise level [114].

Since the soft module in assembled FHEESs does not contain any stiff components, it is possible to create substrate-free soft modules. A coating with specific compositions could be directly sprayed on the skin to form a tightly skin-attached film sensor. Combined with a stiff processing module, a substrate-less assembled FHEES for rapid hand task recognition was realized, which eliminates the motion constraint and achieves meta-learning within the system [30]. Ershad et al. [115] proposed ultra-conformal drawn-on-skin electronics to achieve multifunctional on-skin electronic devices. The drawn-on-skin electronic is created by liquid functional inks drawn into stencils using ballpoint pens directly on human skin. Therefore, on-skin devices form an ultra-conformal, robust, and stretchable interface with the skin, improving the signal quality.

Besides, other types of polymer materials (e.g., styrene ethylene butylene styrene [SBS] and polymethyl methacrylate [PMMA]), emerging hydrogel materials (biocompatibility, excellent skin-conformal capabilities, and adjustable mechanical and electrical properties), etc., are also being explored for the construction of assembled multimodal FHEESs [19,100,116–119]. Some properties of these substrates are listed in Table 2. The structural characteristics of assembled FHEESs, which separate the soft modules from the stiff modules, broaden the substrate selection range for the soft modules.

#### Patterning process

Assembled multimodal FHEESs have various patterning methods, including the photolithography process, printing process, and some special processes for specific substrates, such as laser and electroplating (Fig. 4C).

The photolithography process involves the transfer of the pattern on the mask to the target substrate photoresist with the help of light. With the patterned photoresist, specific patterns can be formed through material deposition/etching or growth processes. At present, the application of photolithography in assembled multimodal FHEESs is generally combined with substrates that are resistant to the photolithography process, such as PI and PET (Table 2), which can process fine patterns (minimum line width <50 μm). A common process is spin-coating the precursor on Si or SiO_2_ wafers and then thermal curing to form the polymer substrate. The metal film is deposited on the PI substrate by thermal evaporation or sputtering. The photolithography process is used to make the photoresist cover the metal pattern that needs to be protected (or before the metal deposition to avoid gaps being filled with metal). Finally, the unprotected metal is removed by etching, and patterned metal interconnects or electrodes are obtained [6].

The printing process is a time-honored patterning method and is suitable for the preparation of functional patterns on paper or plastic substrates. In assembled multimodal FHEESs, spraying, scraping, dipping, screen printing, transfer, and other methods have been used. Their common characteristics are low cost, simple, and efficient operation [21,66,112,116,120,121]. The roll-to-roll manufacturing process is a printing method that can achieve scale manufacturing. Khuje et al. [122] used a compatible extrusion-based direct writing technique, followed by the lamination of the polycarbonate film by hot-pressing to realize the roll-to-roll manufacture of multimodal pressure and flow sensing devices.

Laser patterning is an efficient and precise method. The process of laser reduction of graphene oxide (GO) can selectively reduce to form graphene on any substrate coated with GO solution [45,123,124]. Laser engraving can selectively remove unwanted materials to form specific pattern structures [44,125], and the 3D processing capability of laser provides a solution for preparing multimodal integrated sensors with complex layered structures [126].

The electroplating process is completed on the conductor substrate, thereby limiting the application scope. However, there are also examples where researchers have formed a patterned conductive pattern on an insulating substrate and then electroplated to obtain the desired metal pattern. It is noteworthy that this method can be used for the preparation of soft modules based on LM. Zhuang et al. presented an ingenious process for preparing LM patterns on the fiber film substrates. They prepared Ag microelectrodes on a SiO_2_ wafer coated with dextran and then proceeded to form the SBS fiber mat directly on the Ag patterns by electrospinning. After dissolving the dextran layer, the Ag microelectrodes were transferred to the SBS fiber mat. Finally, the fiber mats were wetted with LM. Because of the poor affinity to SBS and high reactivity with Ag, LM (EGaIn) selectively dewets from the SBS surface and wets on the Ag-covered areas to form the designed LM patterns [127].

#### Stretchable conductor

Stretchable conductors can fully exploit their advantage of soft and thin in the soft module of assembled FHEESs. Due to their intrinsic elastic property, stretchable conductors will not introduce soft–stiff interfacial instability in soft modules. At present, stretchable conductors can be divided into 3 categories: carbon-based, nano-metal-based, and LMs (Fig. 4D).

The research of carbon-based composite conductors started early, and it is generally obtained by mixing low-dimensional carbon materials, such as carbon black, carbon nanotubes (CNTs), and graphite, into the elastic matrix; thus, it balances both stretchability and electrical conductivity [128,129]. Kim et al. achieved stretchable and conductive electrodes based on hybrid carbon nanocomposites. Both CNTs and graphite were added into the PDMS precursor to build the more effective conductive microchannels, and gecko-inspired micropatterns were formed by reverse molding technology to provide dry adhesive characteristics [90]. However, it is challenging to improve the conductivity of carbon-based stretchable conductors; thus, composites filled with metal nanomaterials with higher electrical conductivity have emerged [120,130–132]. Because the microscopic connection formed by the nanomaterials determines the conductivity, the limitation of composite-based stretchable conductors lies in that their conductivity will change with strain (strain causes the change of microscopic connection). This non-ideal interconnect property hinders their further use.

LM with high conductivity can maintain fluidity at or near room temperature. Mercury is the most familiar LM, but it is toxic [133]. At present, non-toxic or low-toxicity LMs, mainly represented by eutectic gallium-indium (EGaIn), have been widely used in FHEESs, which can not only provide ultra-high conductivity but also realize excellent stretchability as flexible interconnects or electrodes [126,134]. Directly using EGaIn to prepare stretchable interconnects or electrodes are challenging due to its difficulty in patterning. Consequently, it is often dispersed into small particle coatings through a pretreatment process. For example, bulk LM and polystyrene sulfonate were added in diluted acetic acid and were tip-sonicated to prepare the LM ink for printing. In this way, LM can be printed on the target substrate and form robust adhesion with the substrate [135]. However, the biocompatibility of LM still needs to be further studied, and the leakage issue caused by fluidity remains to be resolved. In addition, researchers should pay more attention to the patterning process of LM and the cost reduction issue.

### Applied field and advantages

#### Limb-end sensing

**Fig. 5.**
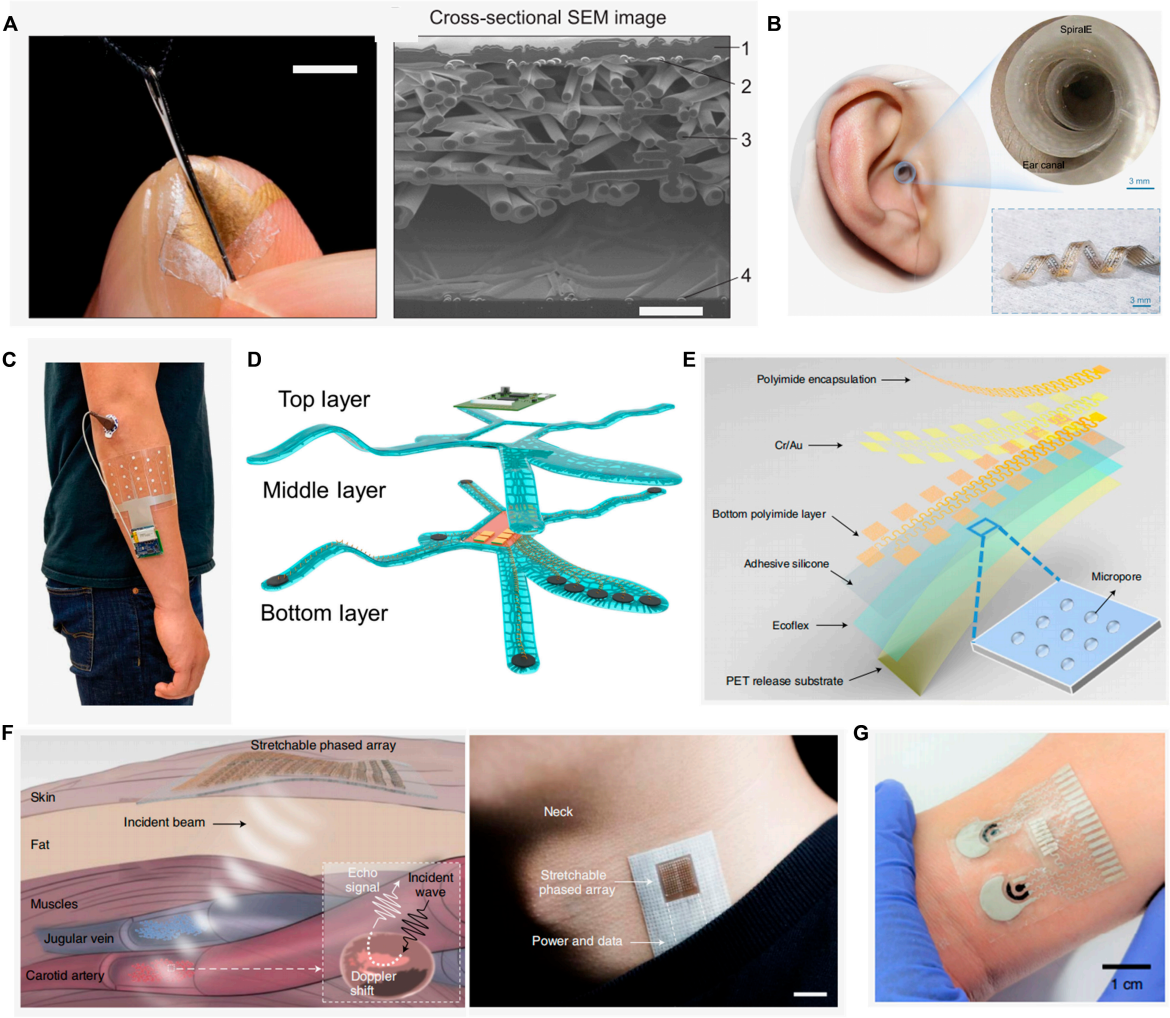
Applications for assembled multimodal FHEESs. (A) Assembled multimodal FHEESs based on the nanomesh pressure sensor for monitoring finger manipulation without sensory interference, composed of a four-layer fiber mesh structure. Copyright 2020, AAAS, reproduced with permission [136]. (B) Assembled FHEESs achieve conformal in-ear bioelectronics for brain-computer interfaces. Reprinted/Adapted from Wang et al [118]. (C) A gesture recognition assembled FHEES based on multi-channel EMG of the forearm. Copyright 2020, Springer Nature, reproduced with permission [32]. (D) With integrated thin-film electrodes and interconnects as the soft module, assembled FHEESs can acquire epidermal electrophysiology at scale. Copyright 2023, Wiley, reproduced with permission [66]. (E) The layered structure diagram for large-area MRI (Magnetic Resonance Imaging)-compatible epidermal electronic interfaces for prosthetic control and cognitive monitoring. Copyright 2019, Springer Nature, reproduced with permission [139]. (F) The blood flow monitoring principles and photos of an assembled FHEES with stretchable ultrasonic phased arrays as the soft module. Copyright 2021, Springer Nature, reproduced with permission [141]. (G) The image of an epidermal patch for simultaneously monitoring hemodynamic and metabolic biomarkers. Copyright 2021, Springer Nature, reproduced with permission [144].

Limb-end sensing refers to the sensing and feedback technology for the ends of limbs far away from the trunk, such as fingertips, palms, feet, and ankles. These parts are the front end of human interaction with the outside world, and the traditional cumbersome and rigid equipment will hinder their function. Taking the fingertips as an example, for sensing or tactile feedback on the fingertip, wearing rigid devices or circuits will completely isolate the fingertips from the outside world. Therefore, from the perspective of comfort and measurement accuracy, past electronic systems cannot collect information or give feedback to the fingertip without sensory interference. Due to the complete separation of soft and stiff modules, the soft modules in assembled FHEESs can achieve ultimate softness and thinness (stretchability > 20%, Young’s modulus < 1 MPa, and thickness < 50 μm). Therefore, assembled FHEESs are the most promising technological means to solve application issues of limb-end sensing. A classic study is a nanomesh pressure sensor for monitoring finger manipulation presented by the Someya group. The sensor adopts a 4-layer film structure, composed of top and bottom Au nanomesh layers, a perylene-coated polyurethane nanomesh intermediate layer, and a polyurethane nanomesh-embedded passivation layer. All the layers are manufactured through electrospinning; thus, the characteristics of breathability (nanomesh-based) and thinness (~15 μm) make the sensor perfectly attached to the finger without detectable effects on human sensation (Fig. 5A) [136].

The special position of the palm, foot, or face determines that traditional rigid devices cannot be placed for a long time (especially under motion) [30,105,106]. The assembled FHEESs allow soft modules to be placed at these sensitive locations, while the stiff modules are positioned away from the limb end. An interesting application is the in-ear brain–computer interface system. Conventional non-invasive brain–computer interface devices (such as headband-based commercial products or microneedle-based approaches) collect bioelectronic signals from the brain, but their wide use is limited by their low signal quality, high inflammation risk, unaesthetic appearance, and inconvenient use. The ear canal is close to the brain, and the environment inside the ear is more stable and reliable than on the body surface, which is an ideal hidden location for collecting electrical signals from the brain. However, the space inside the ear is narrow, and the in-ear skin is sensitive and fragile, thus relying on rigid devices to insert the ear will cause an uncomfortable wearing experience. Based on assembled FHEESs, in-ear soft bioelectronics (the soft module) can adaptively expand and spiral along the auditory meatus under electrothermal actuation, thus ensuring conformal contact while avoiding over-constraining the meatus (Fig. 5B) [118].

#### Multichannel physiological electrical signal acquisition

The measurement of electrophysiological signals often requires parallel acquisition of multiple channels placed at specific locations to obtain effective and comprehensive information. Therefore, the area of the epidermal electronic system for the acquisition of multi-channel electrophysiological signals is usually large, which is suitable for assembled structures.

sEMG can be used to identify and predict human motion patterns in human–machine interaction. However, the information provided by single-channel sEMG is limited, and a multi-channel EMG acquisition system is the inevitable development trend. With the assembled FHEES, soft modules containing multi-channel sEMG acquisition electrodes can cover a certain area of the human skin (such as the arm), providing sufficient physiological data information for motion pattern recognition (Fig. 5C) [32]. Besides, sEMG can be used in non-invasive and targeted therapeutic interventions for patients with movement disorders. The conventional rigid metal electrodes with conductive gels and aggressive adhesives can be replaced by breathable, large-area epidermal electronic systems, which achieved ~200 kPa modulus, clothing-like breathability, and 16-channel lightweight wearing experiences [137].

The electrical signal generated by the heart activity is transmitted to the human skin through body fluids and collected by the measurement system to obtain the visual ECG waveforms. A single-lead ECG is an ECG signal obtained from 2 electrode points, which can only reflect the activity state of the heart in one direction. Therefore, it can only provide an approximate estimation of heart rate and a few arrhythmia diseases. The standard 12-lead ECG is the gold standard used for medical diagnosis [138]. The electrode positions in the 12-lead ECG are strictly regulated to reflect the motion of the heart from different directions. They are located all over the chest and limbs, and only the assembled FHEESs can provide such a large area of soft sensing modules (Fig. 5D) [31,66]. Tian et al. presented a manufacturing approach to process large-area epidermal electronic interfaces. They demonstrated the use of the proposed devices with 8 pairs of electrodes to provide 8 channels of bipolar EMG for prosthetic control. The electrical impedance of the mesh flexible electrodes is similar to that of the traditional gel-based wet electrodes, which are most relevant to physiological electrical signal acquisition. Besides, they used this device to acquire EEG/ECG data from a patient inside an MRI scanner to showcase the convenience of large-area epidermal electronic interfaces in hospital settings (Fig. 5E) [139].

In addition, the acquisition of surface EEG and electrooculogram (EOG) signals using assembled FHEESs also paves the way for novel modes of human–machine interaction. The more channels the EEG has, the more content it can recognize. Limited to the presence of scalp hair in the head, the assembled FHEES also explores the aforementioned in-ear brain–computer interface system [118]. If only a single-channel EOG signal is collected, only one-dimensional control can be achieved, while multichannel EOG can achieve multi-dimensional human–machine interaction. Based on the assembled FHEESs with skin-like soft modules (<1 MPa), thinness (<50 μm), and electrical impedance similar to wet electrodes, the human–machine interaction using EEG or EOG will be more comfortable and accurate, and due to the skin-conformal capability, assembled FHEESs promise more stable dynamic acquisition [7,94,140].

For the multichannel physiological electrical signal acquisition that adopts the separated system structure, the ultra-thin electrode can effectively promote the conformality between the electrode and the skin, reducing the disturbance caused by strain. However, the impact of strain also involves the influence on the leads and the interface between the leads and the processing circuit board, which would cause signal disturbance or even circuit breaking under strain. The exploration of this problem may start from the perspective of full integration and flexible soft–stiff interfaces [29].

#### Large-area array sensing

Large-area array sensing can be divided into single-type sensor integration and multi-type sensor integration. Because of the large area of the array sensor and the high demand for flexibility, it usually adopts the assembled structure [33].

One typical application of single-type array sensing is the flexible ultrasonic array system. Ultrasonic waves have remarkable penetration depth in the human tissue and provide meaningful information from deep tissues for chronic disease detection. An unfocused single element can sense a region directly beneath it with a penetration depth of ~3 to 4 cm at a spatial resolution of 0.5 mm, but it is difficult to target specific regions with a single element due to the complex human anatomy. The ultrasonic phased array can synchronize an array of transducers to enhance the energy density and enable beam steering (up to 14 cm), which is ideal for deep tissue imaging. Conventional devices are still on the stiff substrate, and require manual holding during measurement, hindering their daily application. Prof. Xu’s group successfully built stretchable ultrasonic phased arrays for continuous monitoring of deep-tissue hemodynamics. The system is composed of a stretchable ultrasonic phased array (soft module) and a control circuit (stiff module). ACF was used to connect the array to the control circuit. The stretchable ultrasonic phased array adopts conductive composites, structural copper stretchable interconnects, piezoelectric units, and other materials to achieve thinness and stretchability. Corresponding experiments conducted by the researchers have proven their efficiency in cardiac activity monitoring, blood flow monitoring, multi-site mapping, and so on (Fig. 5F). However, the beam intensity from the flexible ultrasonic array will be affected by tensile strain. Although a long-wavelength ultrasonic array was adopted to minimize the sensitivity of beamforming to shape changes of the device, its effective usage range is still limited within ±20% tensile strain [141,142].

The integration of multi-type sensors is commonly used in assembled multimodal FHEESs for synthetic analysis. He et al. proposed an integrated textile sensor patch for real-time and multiplex sweat analysis. The silk fabric-derived carbon textile was selected as the substrate and used as the working electrode base for electrochemical sensing, which provides rich active sites, and good water wettability. It is capable of simultaneous and highly sensitive detection of glucose (a concentration range of 25 to 300 μM, a sensitivity of 6.3 nA μM^−1^, and a limit of detection of 5 μM), lactate (a linear range of 5 to 35 mM and a limit of detection of 0.5 mM with a sensitivity of 174.0 nA mM^−1^), ascorbic acid (a linear range of 20 to 300 μM with a limit of detection of 1 μM and a sensitivity of 22.7 nA μM^−1^), uric acid (a linear range of 2.5 to 115 μM with a sensitivity of 196.6 nA μM^−1^), Na^+^, and K^+^ (5 to 100 mM for Na+ and 1.25 to 40 mM for K+, a sensitivity of 51.8 and 31.8 mV per decade of concentration for Na+ and K+, respectively; the limits of detection of the Na+ and K+ sensors were 1 and 0.5 mM, respectively) biomarkers. Along with the stiff module (signal collection/transduction/processing, and wireless transmission components), this patch has enabled users to realize real-time analysis of sweat [143]. Besides, monitoring blood pressure, heart rate, hemodynamic parameters, and metabolic indicators in a single system has been presented. The assembled structure of the presented non-invasive skin-worn device ensures mechanical resiliency and flexibility while conforming to curved skin surfaces, and ensures reliable sensing of glucose in ISF and of lactate, caffeine, and alcohol in sweat. Because the ISF sensor, the sweet sensor, and the strain sensor are designed to be placed separately, the crosstalk between them has been suppressed, which was proved by monitoring the changes in one signal while the other signal was generated intermittently (Fig. 5G) [144].

## Discussion

The advent of multimodal FHEESs promises to revolutionize healthcare and human–machine interaction. According to the different system structures, they can be divided into 2 categories: all-in-one and assembled.

Their differences are reflected in aspects of manufacture, structures, properties, and performance. All-in-one FHEESs place stiff components on elastic or flexible substrates and interconnections; thus, flexible layer manufacturing and stiff component implantation often require alternating processes. This leads to the coupling of soft and stiff components in the all-in-one FHEES. Although the island-bridge structure can partially solve the mechanical incompatibility between soft and stiff components, it still results in the non-ideal flexibility of all-in-one FHEESs. Therefore, the performance of all-in-one FHEES in applications that require well-dynamic skin conformality for accurate signal collection is not satisfactory. However, the vertical structure and many traditional processes (such as laser cutting and welding) used in the manufacturing process make all-in-one FHEESs’ integration and stability comparable to FPCBs in non-strain conditions. Their high-integrated structure allows them to reduce the contact area with the skin to minimize strain effects, which determines their advantageous position in small-area epidermal applications (such as motion detection for HMI, body fluid analysis, and acoustic monitoring as mentioned above). Assembled FHEESs adopt a separate structure for soft modules and stiff modules, with a special soft–stiff interface to connect them. Because both the design and manufacture are separated for soft modules and stiff modules, the soft module in assembled FHEESs can adopt special manufacturing techniques (such as skin drawing and electrospinning) to form functional devices with substrate-free or ultra-thin structures. Without the constraints of stiff components, it is easy to prepare large-area, skin-conformal, and sensation-free soft modules (for example, thickness < 50 μm, modulus < 2 MPa, and stretchability > 30%), thus promising applications that require multi-point measurement, high stretchability, or high comfort.

As mentioned above, the all-in-one FHEESs offer high integration and brilliant stability, while the assembled FHEESs have large areas and superior skin conformality. Therefore, they are suitable for different application scopes and face distinct challenges in design and use.

For all-in-one multimodal FHEESs, the following challenges must be addressed:

1. Delamination. The heterogeneous integration of flexible devices and rigid components in all-in-one multimodal FHEESs results in a mismatch in mechanical properties, leading to delamination and significantly reducing the reliability of all-in-one multimodal FHEESs.
2. Skin conformality. The incorporation of rigid components within the design of all-in-one multimodal FHEESs presents a challenge in achieving conformal adhesion with the skin, which is essential for the acquisition of high-quality signals. This is due to the limited stretchability and thinness of the skin, which are incompatible with the stiffness of the devices.
3. Energy source. Achieving high-efficiency wireless power or self-power sources within the all-in-one FHEESs will greatly reduce the mass and volume, thereby improving comfort and application scopes.
4. Permeability. The stacked structure of all-in-one FHEESs impedes the skin’s breathing and prevents the system from being used for a long time. Therefore, it is meaningful to develop porous/permeable all-in-one multimodal FHEESs.
5. Data transmission. Signal wireless transmission is a significant source of power consumption, particularly in multimodal sensing and real-time monitoring applications. Local processing can further enhance the operation lifespan of the system.
6. Manufacturing process. The integrated manufacturing of soft and rigid devices presents unique challenges with no efficient and universal manufacturing process reported. Existing all-in-one FHEESs rely on manual operation in the laboratory, limiting their scalability and utility.

For assembled multimodal FHEESs, challenging issues lie in the following:

1. Soft–stiff interfaces. The key to forming the functional system of assembled FHEESs is the realization of soft–stiff interfaces. However, the research on soft–stiff interfaces is still in its infancy. A reliable soft–stiff interface that can complete the assembly of soft and stiff modules at any time and in any location should possess the following characteristics: a stable electrical connection can be formed with both soft and stiff modules, universality, stretchability, low cost, and a simple manufacturing process.
2. Stability. Stability is an important issue for assembled multimodal FHEESs due to the flexibility of soft modules and differences in users’ skin. Further research is required to develop effective packaging strategies, encapsulation materials, and calibration techniques that remain to be further explored.
3. Selectivity. Selectivity is a major concern for mechanical sensing and electrochemical analysis. For example, pressure and strain can interfere with each other, and temperature can affect the sensor’s response to biomarkers in sweat. Potential solutions may involve the customization of the soft module structure for the specific application scenario and the assistance of calibration hardware or software.
4. Recyclability. Soft modules in assembled multimodal FHEESs are typically disposable, necessitating the development of recycling technology for soft modules in FHEESs to reduce resource consumption and manufacturing costs.

Challenges and opportunities coexist. To continuously promote the development of FHEES, research institutions, and industry should pay attention to the following aspects:

1. Biomimetic multimodal epidermal electronic system. The structure of living organisms often possesses multimodal characteristics, and imitating their construction and integration methods can help achieve a more comprehensive epidermal electronic system. For example, a 3D electronic skin inspired by the spatial distribution of receptors in the skin exhibits excellent mechanical decoupling performance, which can measure pressure, shear force, and strain at the same time. This electronic skin can perceive the curvature and modulus of objects through touch [145].
2. Implementing the human–machine fusion system by soft–stiff interface technology. Integrating electronic devices with the human body through skin films is expected to achieve a new paradigm of enhancing human functions, daily health management, and human–machine collaboration. At present, the operation of flexible thin-film electronic components still requires the coordination of traditional stiff electronic components. There is an urgent need to solve the problem of connecting the on-skin components to devices outside the skin. As mentioned above, a plug-and-play interface can be realized by adjusting the density of gold nanoparticles deposited on an adhesive substrate, which achieves stable, conductive, stretchable, and self-adhesive connections between different components (soft and soft, soft and stiff) [29]. However, the research on developing soft and stiff skin interfaces with lower costs, suitable for different substrates and stable skin adhesion, is still in its early stages.
3. Conductor and semiconductor elastic materials innovation. The development from hybrid electronic systems to fully flexible electronic systems will inevitably replace existing silicon-based chips and printed circuit boards. Finding elastic conductor materials and semiconductor materials with good performance is the foundation for constructing flexible digital gate devices or analog devices. Based on stretchable organic and nanomaterials, intrinsically stretchable diodes capable of operating at a frequency as high as 13.56 MHz have been presented [146]. An e-skin system without rigid electronic components achieves multimodal perception, neuromorphic pulse-train signal generation, and closed-loop actuation. This is because they adopted intrinsic elastic conductors and semiconductor materials, and made unique innovations in the engineering of material properties [147].
4. Innovation and integration of manufacturing processes. There have been many studies on the manufacturing process of hybrid epidermal electronic systems, and some typical processes such as inkjet printing and laser engraving can quickly form flexible electronic components, but the integration process of how flexible electronic devices and traditional electronic devices are assembled to form hybrid electronic systems is not mature. This issue is mainly reflected in many all-in-one FHEES studies where the manual placement of stiff components is still used to assemble their hybrid electronic systems [28,83]. In terms of innovation, 3D manufacturing and manufacturing on the curved surface have attracted a lot of attention. Take the representative of 3D manufacturing technology, 3D printing, as an example; its progress is reflected in incorporating more diverse materials into the machinability range, such as hydrogel and conductive composites [69,148] and more precise control [149]. Besides, for manufacturing on 3D surface, transfer printing, self-assembly, direct writing, and other methods enable the processing of flexible electronic devices on curved surfaces and obtain conformal electronic devices applied to body surfaces [150–152]. Currently, there are still a lot of issues to be explored in terms of consistency between planar and curved shapes, the range of materials that can be manufactured, component adhesion on curved surfaces, etc.
5. Innovation and application of self-power technology. Self-power technology will help epidermal electronic systems achieve long-term operation, broadening their working space. For example, to continuously collect multimodal physicochemical data across indoor and outdoor physical activities for over 12 hours, a hybrid electronic wearable system adopts perovskite solar cells to collect energy from the surrounding light [80]. However, due to the dependence on environmental energy, all existing self-power methods have application limitations. Hybrid energy supply solutions are promising in the future.
6. Collaboration with machine learning/artificial intelligence. Multimodal hybrid flexible electronic systems will bring numerous physiological data around the clock. To save manpower and improve real-time performance, the denoising and analysis of these data require the cooperation of artificial intelligence (the development trend from cloud to edge), which has become a consensus [25,153].

## References

[1] Ma Y, Zhang Y, Cai S, Han Z, Liu X, Wang F, Cao Y, Wang Z, Li H, Chen Y, et al. Flexible hybrid electronics for digital healthcare. Adv Mater. 2020;32(15):1902062. 10.1002/adma.201902062 31243834

[2] Ma S, Kumaresan Y, Dahiya AS, Dahiya R. Ultra-thin chips with printed interconnects on flexible foils. Adv Electron Mater. 2022;8(5):2101029.

[3] Biggs J, Myers J, Kufel J, Ozer E, Craske S, Sou A, Ramsdale C, Williamson K, Price R, White S. A natively flexible 32-bit arm microprocessor. Nature. 2021;595(7868):532–536. 34290427 10.1038/s41586-021-03625-w

[4] Wang S, Xu J, Wang W, Wang GJN, Rastak R, Molina-Lopez F, Chung JW, Niu S, Feig VR, Lopez J, et al. Skin electronics from scalable fabrication of an intrinsically stretchable transistor array. Nature. 2018;555(7694):83–88. 29466334 10.1038/nature25494

[5] Oh H, Oh JY, Park CW, Pi JE, Yang JH, Hwang CS. High density integration of stretchable inorganic thin film transistors with excellent performance and reliability. Nat Commun. 2022;13(1):4963. 36002441 10.1038/s41467-022-32672-8 PMC9402572

[6] Lee H, Song C, Hong YS, Kim M, Cho HR, Kang T, Shin K, Choi SH, Hyeon T, Kim DH. Wearable/disposable sweat-based glucose monitoring device with multistage transdermal drug delivery module. Sci Adv. 2017;3(3): Article e1601314. 28345030 10.1126/sciadv.1601314 PMC5342654

[7] Xu J, Li X, Chang H, Zhao B, Tan X, Yang Y, Tian H, Zhang S, Ren TL. Electrooculography and tactile perception collaborative interface for 3D human-machine interaction. ACS Nano. 2022;16(4):6687–6699. 35385249 10.1021/acsnano.2c01310

[8] Tian H, Li X, Wei Y, Ji S, Yang Q, Gou GY, Wang X, Wu F, Jian J, Guo H, et al. Bioinspired dual-channel speech recognition using graphene-based electromyographic and mechanical sensors. Cell Rep Phys Sci. 2022;3(10): Article 101075.

[9] Xu S, Xu Z, Li D, Cui T, Li X, Yang Y, Liu H, Ren TL. Recent advances in flexible piezoresistive arrays: Materials, design, and applications. Polymers. 2023;15(12):2699. 37376345 10.3390/polym15122699 PMC10304338

[10] Dusek JE, Triantafyllou MS, Lang JH. Piezoresistive foam sensor arrays for marine applications. Sens Actuators A Phys. 2016;248:173–183.

[11] Zheng Y, Liu H, Wang J, Cui T, Zhu J, Gui Z. Unlocking intrinsic conductive dynamics of ionogel microneedle arrays as wearable electronics for intelligent fire safety. Adv Fiber Mater. 2024;6(1):195–213.

[12] Liu H, Zhang H, Ren B, Zheng Y, Cao W, Lu Y, Nie Z, Xu F, Huang W, Zhu J. Robust ionics reinforced fiber as implantable sensor for early operando monitoring cell thermal safety of commercial lithium-ion batteries. Nano Lett. 2024;24(7):2315–2321. 38341875 10.1021/acs.nanolett.3c04709

[13] Nassar JM, Khan SM, Velling SJ, Diaz-Gaxiola A, Shaikh SF, Geraldi NR, Torres Sevilla GA, Duarte CM, Hussain MM. Compliant lightweight non-invasive standalone “Marine Skin” tagging system. Npj Flex Electron. 2018;2:13.

[14] Gu G, Zou J, Zhao R, Zhao X, Zhu X. Soft wall-climbing robots. Sci Robot. 2018;3(25):eaat2874. 33141690 10.1126/scirobotics.aat2874

[15] Luo Y, Abidian MR, Ahn JH, Akinwande D, Andrews AM, Antonietti M, Bao Z, Berggren M, Berkey CA, Bettinger CJ, et al. Technology roadmap for flexible sensors. ACS Nano. 2023;17(6):5211–5295. 36892156 10.1021/acsnano.2c12606 PMC11223676

[16] Yang R, Zhang W, Tiwari N, Yan H, Li T, Cheng H. Multimodal sensors with decoupled sensing mechanisms. Adv Sci. 2022;9(26):2202470. 10.1002/advs.202202470 PMC9475538 35835946

[17] Yang JC, Mun J, Kwon SY, Park S, Bao Z, Park S. Electronic skin: Recent progress and future prospects for skin-attachable devices for health monitoring, robotics, and prosthetics. Adv Mater. 2019;31(48):1904765. 10.1002/adma.201904765 31538370

[18] Yang Y, Cui T, Li D, Ji S, Chen Z, Shao W, Liu H, Ren TL. Breathable electronic skins for daily physiological signal monitoring. Nanomicro Lett. 2022;14(1):161. 35943631 10.1007/s40820-022-00911-8 PMC9362661

[19] Wang L, Xu T, Zhang X. Multifunctional conductive hydrogel-based flexible wearable sensors. TrAC Trends Analyt Chem. 2021;134: Article 116130.

[20] Guo Y, Wei X, Gao S, Yue W, Li Y, Shen G. Recent advances in carbon material-based multifunctional sensors and their applications in electronic skin systems. Adv Funct Mater. 2021;31(40):2104288.

[21] Kim K, Kim B, Lee CH. Printing flexible and hybrid electronics for human skin and eye-interfaced health monitoring systems. Adv Mater. 2020;32(15):1902051. 10.1002/adma.201902051 31298450

[22] Liu E, Cai Z, Ye Y, Zhou M, Liao H, Yi Y. An overview of flexible sensors: Development, application, and challenges. Sensors. 2023;23(2):817. 36679612 10.3390/s23020817 PMC9863693

[23] Lin Y, Bariya M, Javey A. Wearable biosensors for body computing. Adv Funct Mater. 2021;31(39):2008087.

[24] Liu X, Wei Y, Qiu Y. Advanced flexible skin-like pressure and strain sensors for human health monitoring. Micromachines. 2021;12(6):695. 34198673 10.3390/mi12060695 PMC8232132

[25] Ates HC, Nguyen PQ, Gonzalez-Macia L, Morales-Narváez E, Güder F, Collins JJ, Dincer C. End-to-end design of wearable sensors. Nat Rev Mater. 2022;7(11):998–907. 10.1038/s41578-022-00460-x PMC9306444 35910814

[26] Song H, Luo G, Ji Z, Bo R, Xue Z, Yan D, Zhang F, Bai K, Liu J, Cheng X, et al. Highly-integrated, miniaturized, stretchable electronic systems based on stacked multilayer network materials. Sci Adv. 2022;8(11):eabm3785. 35294232 10.1126/sciadv.abm3785 PMC8926335

[27] Ma X, Wang P, Huang L, Ding R, Zhou K, Shi Y, Chen F, Zhuang Q, Huang Q, Lin Y, et al. A monolithically integrated in-textile wristband for wireless epidermal biosensing. Sci Adv. 2023;9(45):eadj2763. 37948514 10.1126/sciadv.adj2763 PMC10637736

[28] Huang Z, Hao Y, Li Y, Hu H, Wang C, Nomoto A, Pan T, Gu Y, Chen Y, Zhang T, et al. Three-dimensional integrated stretchable electronics. Nat Electron. 2018;1(8):473–480.

[29] Jiang Y, Ji S, Sun J, Huang J, Li Y, Zou G, Salim T, Wang C, Li W, Jin H, et al. A universal interface for plug-and-play assembly of stretchable devices. Nature. 2023;614(7948):456–462. 36792740 10.1038/s41586-022-05579-z

[30] Kim KK, Kim M, Pyun K, Kim J, Min J, Koh S, Root SE, Kim J, Nguyen BNT, Nishio Y, et al. A substrate-less nanomesh receptor with meta-learning for rapid hand task recognition. Nat Electron. 2023;6(1):64–75.

[31] Wang Y, Yin L, Bai Y, Liu S, Wang L, Zhou Y, Hou C, Yang Z, Wu H, Ma J, et al. Electrically compensated, tattoo-like electrodes for epidermal electrophysiology at scale. Sci Adv. 2020;6(43):eabd0996. 33097545 10.1126/sciadv.abd0996 PMC7608837

[32] Moin A, Zhou A, Rahimi A, Menon A, Benatti S, Alexandrov G, Tamakloe S, Ting J, Yamamoto N, Khan Y, et al. A wearable biosensing system with in-sensor adaptive machine learning for hand gesture recognition. Nat Electron. 2021;4(1):54–63.

[33] Hua Q, Sun J, Liu H, Bao R, Yu R, Zhai J, Pan C, Wang ZL. Skin-inspired highly stretchable and conformable matrix networks for multifunctional sensing. Nat Commun. 2018;9(1):244. 29339793 10.1038/s41467-017-02685-9 PMC5770430

[34] Xu S, Zhang Y, Jia L, Mathewson KE, Jang KI, Kim J, Fu H, Huang X, Chava P, Wang R, et al. Soft microfluidic assemblies of sensors, circuits, and radios for the skin. Science. 2014;344(6179):70–74. 24700852 10.1126/science.1250169

[35] Li E, Rao Z, Wang X, Liu Y, Yu R, Chen G, Chen H, Guo T. Direct fabrication of stretchable electronics on a programmable stiffness substrate with 100% strain isolation. IEEE Electron Device Lett. 2021;42(10):1484–1487.

[36] Romeo A, Liu Q, Suo Z, Lacour SP. Elastomeric substrates with embedded stiff platforms for stretchable electronics. Appl Phys Lett. 2013;102(13): Article 131904.

[37] Valentine AD, Busbee TA, Boley JW, Raney JR, Chortos A, Kotikian A, Berrigan JD, Durstock MF, Lewis JA. Hybrid 3D printing of soft electronics. Adv Mater. 2017;29(40):1703817. 10.1002/adma.201703817 28875572

[38] Chung HU, Kim BH, Lee JY, Lee J, Xie Z, Ibler EM, Lee K, Banks A, Jeong JY, Kim J, et al. Binodal, wireless epidermal electronic systems with in-sensor analytics for neonatal intensive care. Science. 2019;363(6430):eaau0780. 30819934 10.1126/science.aau0780 PMC6510306

[39] Lee B, Cho H, Jeong S, Yoon J, Jang D, Lee DK, Kim D, Chung S, Hong Y. Stretchable hybrid electronics: Combining rigid electronic devices with stretchable interconnects into high-performance on-skin electronics. J Inf Disp. 2022;23(3):163–184.

[40] Wang M, Wang K, Ma C, Uzabakiriho PC, Chen X, Zhao G. Mechanical gradients enable highly stretchable electronics based on nanofiber substrates. ACS Appl Mater Interfaces. 2022;14(31):35997–36006. 35894160 10.1021/acsami.2c10245

[41] Lin R, Li Y, Mao X, Zhou W, Liu R. Hybrid 3D printing all-in-one heterogenous rigidity assemblies for soft electronics. Adv Mater Technol. 2019;4(12):1900614.

[42] Someya T, Bao Z, Malliaras GG. The rise of plastic bioelectronics. Nature. 2016;540(7633):379–385. 27974769 10.1038/nature21004

[43] Rogers JA, Someya T, Huang Y. Materials and mechanics for stretchable electronics. Science. 2010;327(5973):1603–1607. 20339064 10.1126/science.1182383

[44] Kabiri AS, Ho R, Jang H, Tao L, Wang Y, Wang L, Schnyer DM, Akinwande D, Lu N. Graphene electronic tattoo sensors. ACS Nano. 2017;11(8):7634–7641. 28719739 10.1021/acsnano.7b02182

[45] Qiao Y, Wang Y, Tian H, Li M, Jian J, Wei Y, Tian Y, Wang DY, Pang Y, Geng X, et al. Multilayer graphene epidermal electronic skin. ACS Nano. 2018;12(9):8839–8846. 30040381 10.1021/acsnano.8b02162

[46] Yan Z, Xu D, Lin Z, Wang P, Cao B, Ren H, Song F, Wan C, Wang L, Zhou J, et al. Highly stretchable van der Waals thin films for adaptable and breathable electronic membranes. Science. 2022;375(6583):852–859. 35201882 10.1126/science.abl8941

[47] Kim DH, Lu N, Ma R, Kim YS, Kim RH, Wang S, Wu J, Won SM, Tao H, Islam A, et al. Epidermal electronics. Science. 2011;333(6044):838–843. 21836009 10.1126/science.1206157

[48] Kim Y-S, Mahmood M, Lee Y, Kim NK, Kwon S, Herbert R, Kim D, Cho HC, Yeo W-H. All-in-one, wireless, stretchable hybrid electronics for smart, connected, and ambulatory physiological monitoring. Adv Sci. 2019;6(17):1900939. 10.1002/advs.201900939 PMC6724359 31508289

[49] Yu X, Xie Z, Yu Y, Lee J, Vazquez-Guardado A, Luan H, Ruban J, Ning X, Akhtar A, Li D, et al. Skin-integrated wireless haptic interfaces for virtual and augmented reality. Nature. 2019;575(7783):473–479. 31748722 10.1038/s41586-019-1687-0

[50] Jung YH, Yoo JY, Vázquez-Guardado A, Kim JH, Kim JT, Luan H, Park M, Lim J, Shin HS, Su CJ, et al. A wireless haptic interface for programmable patterns of touch across large areas of the skin. Nat Electron. 2022;5(6):374–385.

[51] Jang KI, Li K, Chung HU, Xu S, Jung HN, Yang Y, Kwak JW, Jung HH, Song J, Yang C, et al. Self-assembled three dimensional network designs for soft electronics. Nat Commun. 2017;8:15894. 28635956 10.1038/ncomms15894 PMC5482057

[52] Kwon K, Kim JU, Deng Y, Krishnan SR, Choi J, Jang H, Lee K, Su CJ, Yoo I, Wu Y, et al. An on-skin platform for wireless monitoring of flow rate, cumulative loss and temperature of sweat in real time. Nat Electron. 2021;4(4):302–312.

[53] Liu Y, Norton JJS, Qazi R, Zou Z, Ammann KR, Liu H, Yan L, Tran PL, Jang KI, Lee JW, et al. Epidermal mechano-acoustic sensing electronics for cardiovascular diagnostics and human-machine interfaces. Sci Adv. 2016;2(11): Article e1601185. 28138529 10.1126/sciadv.1601185 PMC5262452

[54] Kim YS, Mahmood M, Kwon S, Maher K, Kang JW, Yeo WH. Wireless, skin-like membrane electronics with multifunctional ergonomic sensors for enhanced pediatric care. IEEE Trans Biomed Eng. 2020;67(8):2159–2165. 31794383 10.1109/TBME.2019.2956048

[55] Hassan M, Abbas G, Li N, Afzal A, Haider Z, Ahmed S, Xu X, Pan C, Peng Z. Significance of flexible substrates for wearable and implantable devices: Recent advances and perspectives. Adv Mater Technol. 2022;7(3):2100773.

[56] Li H, Ma Y, Huang Y. Material innovation and mechanics design for substrates and encapsulation of flexible electronics: A review. Mater Horiz. 2021;8(2):383–400. 34821261 10.1039/d0mh00483a

[57] Kwak SS, Yoo S, Avila R, Chung HU, Jeong H, Liu C, Vogl JL, Kim J, Yoon HJ, Park Y, et al. Skin-integrated devices with soft, holey architectures for wireless physiological monitoring, with applications in the neonatal intensive care unit. Adv Mater. 2021;33(44):2103974. 10.1002/adma.202103974 34510572

[58] Wang M, Ma C, Uzabakiriho PC, Chen X, Chen Z, Cheng Y, Wang Z, Zhao G. Stencil printing of liquid metal upon electrospun nanofibers enables high-performance flexible electronics. ACS Nano. 2021;15(12):19364–19376. 34783541 10.1021/acsnano.1c05762

[59] Chen X, Wan H, Guo R, Wang X, Wang Y, Jiao C, Sun K, Hu L. A double-layered liquid metal-based electrochemical sensing system on fabric as a wearable detector for glucose in sweat. Microsyst Nanoeng. 2022;8(1):48. 35542049 10.1038/s41378-022-00365-3 PMC9079077

[60] Yang X, Cheng H. Recent developments of flexible and stretchable electrochemical biosensors. Micromachines. 2020;11(3):243. 32111023 10.3390/mi11030243 PMC7143805

[61] Dincer C, Bruch R, Costa-Rama E, Fernández-Abedul MT, Merkoçi A, Manz A, Urban GA, Güder F. Disposable sensors in diagnostics, food, and environmental monitoring. Adv Mater. 2019;31(30):1806739. 10.1002/adma.201806739 31094032

[62] Li T, Liang B, Ye Z, Zhang L, Xu S, Tu T, Zhang Y, Cai Y, Zhang B, Fang L, et al. An integrated and conductive hydrogel-paper patch for simultaneous sensing of chemical-electrophysiological signals. Biosens Bioelectron. 2022;198: Article 113855. 34871834 10.1016/j.bios.2021.113855

[63] Lyu Q, Gong S, Yin J, Dyson JM, Cheng W. Soft wearable healthcare materials and devices. Adv Healthc Mater. 2021;10(17): Article e2100577. 34019737 10.1002/adhm.202100577

[64] Hsu YY, Gonzalez M, Bossuyt F, Axisa F, Vanfleteren J, De Wolf I. The effects of encapsulation on deformation behavior and failure mechanisms of stretchable interconnects. Thin Solid Films. 2011;519(7):2225–2234.

[65] Nie S, Cai M, Wang C, Song J. Fatigue life prediction of serpentine interconnects on soft elastomers for stretchable electronics. J Appl Mech. 2019;87(1): Article 011011.

[66] Li D, Cui T, Jian J, Yan J, Xu J, Li X, Li Z, Yan A, Chen Z, Shao W, et al. Lantern-inspired on-skin helical interconnects for epidermal electronic sensors. Adv Funct Mater. 2023;33(18):2213335.

[67] Xu S, Yan Z, Jang KI, Huang W, Fu H, Kim J, Wei Z, Flavin M, McCracken J, Wang R, et al. Assembly of micro/nanomaterials into complex, three-dimensional architectures by compressive buckling. Science. 2015;347(6218):154–159. 25574018 10.1126/science.1260960

[68] Kim BH, Li K, Kim JT, Park Y, Jang H, Wang X, Xie Z, Won SM, Yoon HJ, Lee G, et al. Three-dimensional electronic microfliers inspired by wind-dispersed seeds. Nature. 2021;597(7877):503–510. 34552257 10.1038/s41586-021-03847-y

[69] Hui Y, Yao Y, Qian Q, Luo J, Chen H, Qiao Z, Yu Y, Tao L, Zhou N. Three-dimensional printing of soft hydrogel electronics. Nat Electron. 2022;5(12):893–903.

[70] Byun J, Oh E, Lee B, Kim S, Lee S, Hong Y. A single droplet-printed double-side universal soft electronic platform for highly integrated stretchable hybrid electronics. Adv Funct Mater. 2017;27(36):1701912.

[71] Li G, Zhang M, Liu S, Yuan M, Wu J, Yu M, Teng L, Xu Z, Guo J, Li G, et al. Three-dimensional flexible electronics using solidified liquid metal with regulated plasticity. Nat Electron. 2023;6(2):154–163.

[72] Xu H, Zheng W, Zhang Y, Zhao D, Wang L, Zhao Y, Wang W, Yuan Y, Zhang J, Huo Z, et al. A fully integrated, standalone stretchable device platform with in-sensor adaptive machine learning for rehabilitation. Nat Commun. 2023;14(1):7769. 38012169 10.1038/s41467-023-43664-7 PMC10682047

[73] Ye C, Wang M, Min J, Tay RY, Lukas H, Sempionatto JR, Li J, Xu C, Gao W. A wearable aptamer nanobiosensor for non-invasive female hormone monitoring. Nat Nanotechnol. 2024;19:330–337. 37770648 10.1038/s41565-023-01513-0 PMC10954395

[74] Yang P, Li J, Lee SW, Fan HJ. Printed zinc paper batteries. Adv Sci. 2022;9(2):2103894. 10.1002/advs.202103894 PMC8760176 34741445

[75] Rahmanudin A, Khan Z, Tybrandt K, Kim N. Sustainable stretchable batteries for next-generation wearables. J Mater Chem A. 2023;11(42):22718–22736.

[76] Nusrat T, Roy S, Lotfi-Neyestanak AA, Noghanian S. Far-field wireless power transfer for the internet of things. Electronics. 2023;12(1):207.

[77] Fan FR, Tian ZQ, Wang ZL. Flexible triboelectric generator. Nano Energy. 2012;1(2):328–334.

[78] Seung W, Gupta MK, Lee KY, Shin KS, Lee JH, Kim TY, Kim S, Lin J, Kim JH, Kim SW. Nanopatterned textile-based wearable triboelectric nanogenerator. ACS Nano. 2015;9(4):3501–3509. 25670211 10.1021/nn507221f

[79] Song Y, Min J, Yu Y, Wang H, Yang Y, Zhang H, Gao W. Wireless battery-free wearable sweat sensor powered by human motion. Sci Adv. 2020;6(40):eaay9842. 32998888 10.1126/sciadv.aay9842 PMC7527225

[80] Min J, Demchyshyn S, Sempionatto JR, Song Y, Hailegnaw B, Xu C, Yang Y, Solomon S, Putz C, Lehner LE, et al. An autonomous wearable biosensor powered by a perovskite solar cell. Nat Electron. 2023;6(8):630–641. 38465017 10.1038/s41928-023-00996-y PMC10923186

[81] Kwon YT, Kim YS, Kwon S, Mahmood M, Lim HR, Park SW, Kang SO, Choi JJ, Herbert R, Jang YC, et al. All-printed nanomembrane wireless bioelectronics using a biocompatible solderable graphene for multimodal human-machine interfaces. Nat Commun. 2020;11(1):3450. 32651424 10.1038/s41467-020-17288-0 PMC7351733

[82] Tan P, Han X, Zou Y, Qu X, Xue J, Li T, Wang Y, Luo R, Cui X, Xi Y, et al. Self-powered gesture recognition wristband enabled by machine learning for full keyboard and multicommand input. Adv Mater. 2022;34(21): Article e2200793. 35344226 10.1002/adma.202200793

[83] Huang Y, Zhou J, Ke P, Guo X, Yiu CK, Yao K, Cai S, Li D, Zhou Y, Li J, et al. A skin-integrated multimodal haptic interface for immersive tactile feedback. Nat Electron. 2023;6(12):1020–1031.

[84] Steed A, Friston S, Pawar V, Swapp D. Docking haptics: Extending the reach of haptics by dynamic combinations of grounded and worn devices. In *Proceedings of the 26th ACM Symposium on Virtual Reality Software and Technology* (*VRST ‘20)*. New York (NY): Association for Computing Machinery; 2020. p. 1–11.

[85] De Paolis LT, De Luca V. The impact of the input interface in a virtual environment: The vive controller and the myo armband. Virtual Real. 2020;24(3):483–502.

[86] Sempionatto JR, Lasalde-Ramírez JA, Mahato K, Wang J, Gao W. Wearable chemical sensors for biomarker discovery in the omics era. Nat Rev Chem. 2022;6(12):899–915. 37117704 10.1038/s41570-022-00439-w PMC9666953

[87] Min J, Tu J, Xu C, Lukas H, Shin S, Yang Y, Solomon SA, Mukasa D, Gao W. Skin-interfaced wearable sweat sensors for precision medicine. Chem Rev. 2023;123(8):5049–5138. 36971504 10.1021/acs.chemrev.2c00823 PMC10406569

[88] Yang Q, Jin W, Zhang Q, Wei Y, Guo Z, Li X, Yang Y, Luo Q, Tian H, Ren TL. Mixed-modality speech recognition and interaction using a wearable artificial throat. Nat Mach Intell. 2023;5(2):169–180.

[89] Jeong H, Lee JY, Lee K, Kang YJ, Kim JT, Avila R, Tzavelis A, Kim J, Ryu H, Kwak SS, et al. Differential cardiopulmonary monitoring system for artifact-canceled physiological tracking of athletes, workers, and COVID-19 patients. Sci Adv. 2021;7(20):eabg3092. 33980495 10.1126/sciadv.abg3092 PMC8115927

[90] Kim T, Park J, Sohn J, Cho D, Jeon S. Bioinspired, highly stretchable, and conductive dry adhesives based on 1D-2D hybrid carbon nanocomposites for all-in-one ECG electrodes. ACS Nano. 2016;10(4):4770–4778. 26986477 10.1021/acsnano.6b01355

[91] Yamada T, Hayamizu Y, Yamamoto Y, Yomogida Y, Izadi-Najafabadi A, Futaba DN, Hata K. A stretchable carbon nanotube strain sensor for human-motion detection. Nat Nanotechnol. 2011;6(5):296–301. 21441912 10.1038/nnano.2011.36

[92] Lu N, Lu C, Yang S, Rogers J. Highly sensitive skin-mountable strain gauges based entirely on elastomers. Adv Funct Mater. 2012;22(19):4044–4050.

[93] Park S, Kim H, Vosgueritchian M, Cheon S, Kim H, Koo JH, Kim TR, Lee S, Schwartz G, Chang H, et al. Stretchable energy-harvesting tactile electronic skin capable of differentiating multiple mechanical stimuli modes. Adv Mater. 2014;26(43):7324–7332. 25256696 10.1002/adma.201402574

[94] Qiao Y, Li X, Wang J, Ji S, Hirtz T, Tian H, Jian J, Cui T, Dong Y, Xu X, et al. Intelligent and multifunctional graphene nanomesh electronic skin with high comfort. Small. 2022;18(7):2104810. 10.1002/smll.202104810 34882950

[95] Park J, Lee Y, Hong J, Ha M, Jung YD, Lim H, Kim SY, Ko H. Giant tunneling piezoresistance of composite elastomers with interlocked microdome arrays for ultrasensitive and multimodal electronic skins. ACS Nano. 2014;8(5):4689–4697. 24592988 10.1021/nn500441k

[96] Wang X, Gu Y, Xiong Z, Cui Z, Zhang T. Silk-molded flexible, ultrasensitive, and highly stable electronic skin for monitoring human physiological signals. Adv Mater. 2014;26(9):1336–1342. 24347340 10.1002/adma.201304248

[97] Gong S, Schwalb W, Wang Y, Chen Y, Tang Y, Si J, Shirinzadeh B, Cheng W. A wearable and highly sensitive pressure sensor with ultrathin gold nanowires. Nat Commun. 2014;5(1):3132. 24495897 10.1038/ncomms4132

[98] Kang D, Pikhitsa PV, Choi YW, Lee C, Shin SS, Piao L, Park B, Suh KY, Ti K, Choi M. Ultrasensitive mechanical crack-based sensor inspired by the spider sensory system. Nature. 2014;516(7530):222–226. 25503234 10.1038/nature14002

[99] Hozumi S, Honda S, Arie T, Akita S, Takei K. Multimodal wearable sensor sheet for health-related chemical and physical monitoring. ACS Sens. 2021;6(5):1918–1924. 33876933 10.1021/acssensors.1c00281

[100] Song Y, Tay RY, Li J, Xu C, Min J, Shirzaei Sani E, Kim G, Heng W, Kim I, Gao W. 3D-printed epifluidic electronic skin for machine learning-powered multimodal health surveillance. Sci Adv. 2023;9(37):eadi6492. 37703361 10.1126/sciadv.adi6492 PMC10499321

[101] Song E, Chiang CH, Li R, Jin X, Zhao J, Hill M, Xia Y, Li L, Huang Y, Won SM, et al. Flexible electronic/optoelectronic microsystems with scalable designs for chronic biointegration. Proc Natl Acad Sci USA. 2019;116(31):15398–15406. 31308234 10.1073/pnas.1907697116 PMC6681732

[102] Lin YC, Zhong J. A review of the influencing factors on anisotropic conductive adhesives joining technology in electrical applications. J Mater Sci. 2008;43(9):3072–3093.

[103] Kang M, Jeong H, Park SW, Hong J, Lee H, Chae Y, Yang S, Ahn JH. Wireless graphene-based thermal patch for obtaining temperature distribution and performing thermography. Sci Adv. 2022;8(15):eabm6693. 35417247 10.1126/sciadv.abm6693 PMC9007510

[104] Tchoe Y, Bourhis AM, Cleary DR, Stedelin B, Lee J, Tonsfeldt KJ, Brown EC, Siler DA, Paulk AC, Yang JC, et al. Human brain mapping with multithousand-channel PtNRGrids resolves spatiotemporal dynamics. Sci Transl Med. 2022;14(628):eabj1441. 35044788 10.1126/scitranslmed.abj1441 PMC9650779

[105] Jang H, Sel K, Kim E, Kim S, Yang X, Kang S, Ha KH, Wang R, Rao Y, Jafari R, et al. Graphene e-tattoos for unobstructive ambulatory electrodermal activity sensing on the palm enabled by heterogeneous serpentine ribbons. Nat Commun. 2022;13(1):6604. 36329038 10.1038/s41467-022-34406-2 PMC9633646

[106] Kim T, Shin Y, Kang K, Kim K, Kim G, Byeon Y, Kim H, Gao Y, Lee JR, Son G, et al. Ultrathin crystalline-silicon-based strain gauges with deep learning algorithms for silent speech interfaces. Nat Commun. 2022;13(1):5815. 36192403 10.1038/s41467-022-33457-9 PMC9530138

[107] Gao W, Emaminejad S, Nyein HYY, Challa S, Chen K, Peck A, Fahad HM, Ota H, Shiraki H, Kiriya D, et al. Fully integrated wearable sensor arrays for multiplexed in situ perspiration analysis. Nature. 2016;529(7587):509–514. 26819044 10.1038/nature16521 PMC4996079

[108] Shi Y, Yang P, Lei R, Liu Z, Dong X, Tao X, Chu X, Wang ZL, Chen X. Eye tracking and eye expression decoding based on transparent, flexible and ultra-persistent electrostatic interface. Nat Commun. 2023;14(1):3315. 37286541 10.1038/s41467-023-39068-2 PMC10247702

[109] Son J, Bae GY, Lee S, Lee G, Kim SW, Kim D, Chung S, Cho K. Cactus-spine-inspired sweat-collecting patch for fast and continuous monitoring of sweat. Adv Mater. 2021;33(40):2102740. 10.1002/adma.202102740 34396596

[110] Bariya M, Nyein HYY, Javey A. Wearable sweat sensors. Nat Electron. 2018;1(3):160–171.

[111] Wang Y, Yokota T, Someya T. Electrospun nanofiber-based soft electronics. NPG Asia Mater. 2021;13(1):22.

[112] Fan YJ, Li X, Kuang SY, Zhang L, Chen YH, Liu L, Zhang K, Ma SW, Liang F, Wu T, et al. Highly robust, transparent, and breathable epidermal electrode. ACS Nano. 2018;12(9):9326–59332. 30118595 10.1021/acsnano.8b04245

[113] Wang Y, Lee S, Wang H, Jiang Z, Jimbo Y, Wang C, Wang B, Kim JJ, Koizumi M, Yokota T, et al. Robust, self-adhesive, reinforced polymeric nanofilms enabling gas-permeable dry electrodes for long-term application. Proc Natl Acad Sci USA. 2021;118(38): Article e2111904118. 34518214 10.1073/pnas.2111904118 PMC8463786

[114] Xu Y, Guo W, Zhou S, Yi H, Yang G, Mei S, Zhu K, Wu H, Li Z. Bioinspired perspiration-wicking electronic skins for comfortable and reliable multimodal health monitoring. Adv Funct Mater. 2022;32(23):2200961.

[115] Ershad F, Thukral A, Yue J, Comeaux P, Lu Y, Shim H, Sim K, Kim NI, Rao Z, Guevara R, et al. Ultra-conformal drawn-on-skin electronics for multifunctional motion artifact-free sensing and point-of-care treatment. Nat Commun. 2020;11(1):3823. 32732934 10.1038/s41467-020-17619-1 PMC7393123

[116] Imani S, Bandodkar AJ, Mohan AMV, Kumar R, Yu S, Wang J, Mercier PP. A wearable chemical-electrophysiological hybrid biosensing system for real-time health and fitness monitoring. Nat Commun. 2016;7:11650. 27212140 10.1038/ncomms11650 PMC4879240

[117] Hattori Y, Falgout L, Lee W, Jung SY, Poon E, Lee JW, Na I, Geisler A, Sadhwani D, Zhang Y, et al. Multifunctional skin-like electronics for quantitative, clinical monitoring of cutaneous wound healing. Adv Healthc Mater. 2014;3(10):1597–1607. 24668927 10.1002/adhm.201400073 PMC4177017

[118] Wang Z, Shi N, Zhang Y, Zheng N, Li H, Jiao Y, Cheng J, Wang Y, Zhang X, Chen Y, et al. Conformal in-ear bioelectronics for visual and auditory brain-computer interfaces. Nat Commun. 2023;14(1):4213. 37452047 10.1038/s41467-023-39814-6 PMC10349124

[119] Liu H, Xiang H, Wang Y, Li Z, Qian L, Li P, Ma Y, Zhou H, Huang W. A flexible multimodal sensor that detects strain, humidity, temperature, and pressure with carbon black and reduced graphene oxide hierarchical composite on paper. ACS Appl Mater Interfaces. 2019;11(43):40613–40619. 31588725 10.1021/acsami.9b13349

[120] Matsuhisa N, Inoue D, Zalar P, Jin H, Matsuba Y, Itoh A, Yokota T, Hashizume D, Someya T. Printable elastic conductors by in situ formation of silver nanoparticles from silver flakes. Nat Mater. 2017;16(8):834–840. 28504674 10.1038/nmat4904

[121] Cui T, Qiao Y, Li D, Huang X, Yang L, Yan A, Chen Z, Xu J, Tan X, Jian J, et al. Multifunctional, breathable MXene-PU mesh electronic skin for wearable intelligent 12-lead ECG monitoring system. Chem Eng J. 2023;455: Article 140690.

[122] Khuje S, Islam A, Yu J, Ren S. Printing conformal and flexible copper networks for multimodal pressure and flow sensing. Nanoscale. 2023;15(46):18660–18666. 37916506 10.1039/d3nr03481j

[123] Liu H, Xiang H, Li Z, Meng Q, Li P, Ma Y, Zhou H, Huang W. Flexible and degradable multimodal sensor fabricated by transferring laser-induced porous carbon on starch film. ACS Sustain Chem Eng. 2020;8(1):527–533.

[124] El-Kady MF, Strong V, Dubin S, Kaner RB. Laser scribing of high-performance and flexible graphene-based electrochemical capacitors. Science. 2012;335(6074):1326. 22422977 10.1126/science.1216744

[125] Zhao G, Ling Y, Su Y, Chen Z, Mathai CJ, Emeje O, Brown A, Alla DR, Huang J, Kim C, et al. Laser-scribed conductive, photoactive transition metal oxide on soft elastomers for Janus on-skin electronics and soft actuators. Sci Adv. 2022;8(25):eabp9734. 35731865 10.1126/sciadv.abp9734 PMC9216520

[126] Wang B, Prasad S, Hellman O, Li H, Fridberger A, Hjort K. Liquid metal-based high-density interconnect technology for stretchable printed circuits. Adv Funct Mater. 2023;2023:2309707.

[127] Zhuang Q, Yao K, Wu M, Lei Z, Chen F, Li J, Mei Q, Zhou Y, Huang Q, Zhao X, et al. Wafer-patterned, permeable, and stretchable liquid metal microelectrodes for implantable bioelectronics with chronic biocompatibility. Sci Adv. 2023;9(22):eadg8602. 37256954 10.1126/sciadv.adg8602 PMC10413659

[128] Brun M, Chateaux JF, Deman AL, Pittet P, Ferrigno R. Nanocomposite carbon-PDMS material for chip-based electrochemical detection. Electroanalysis. 2011;23(2):321–324.

[129] Sekitani T, Noguchi Y, Hata K, Fukushima T, Aida T, Someya T. A rubberlike stretchable active matrix using elastic conductors. Science. 2008;321(5895):1468–1472. 18687922 10.1126/science.1160309

[130] Choi S, Han SI, Jung D, Hwang HJ, Lim C, Bae S, Park OK, Tschabrunn CM, Lee M, Bae SY, et al. Highly conductive, stretchable and biocompatible Ag–Au core–sheath nanowire composite for wearable and implantable bioelectronics. Nat Nanotechnol. 2018;13(11):1048–1056. 30104619 10.1038/s41565-018-0226-8

[131] Kim K, Kim HJ, Zhang H, Park W, Meyer D, Kim MK, Kim B, Park H, Xu B, Kollbaum P, et al. All-printed stretchable corneal sensor on soft contact lenses for noninvasive and painless ocular electrodiagnosis. Nat Commun. 2021;12(1):1544. 33750806 10.1038/s41467-021-21916-8 PMC7943761

[132] Peng S, Yu Y, Wu S, Wang CH. Conductive polymer nanocomposites for stretchable electronics: Material selection, design, and applications. ACS Appl Mater Interfaces. 2021;13(37):43831–43854. 34515471 10.1021/acsami.1c15014

[133] Dickey MD. Stretchable and soft electronics using liquid metals. Adv Mater. 2017;29(27):1606425. 10.1002/adma.201606425 28417536

[134] Lee W, Kim H, Kang I, Park H, Jung J, Lee H, Park H, Park JS, Yuk JM, Ryu S, et al. Universal assembly of liquid metal particles in polymers enables elastic printed circuit board. Science. 2022;378(6620):637–641. 36356149 10.1126/science.abo6631

[135] Lee GH, Lee YR, Kim H, Kwon DA, Kim H, Yang C, Choi SQ, Park S, Jeong JW, Park S. Rapid meniscus-guided printing of stable semi-solid-state liquid metal microgranular-particle for soft electronics. Nat Commun. 2022;13(1):2643. 35551193 10.1038/s41467-022-30427-z PMC9098628

[136] Lee S, Franklin S, Hassani FA, Yokota T, Nayeem MOG, Wang Y, Leib R, Cheng G, Franklin DW, Someya T. Nanomesh pressure sensor for monitoring finger manipulation without sensory interference. Science. 2020;370(6519):966. 33214278 10.1126/science.abc9735

[137] Kwon YT, Norton JJS, Cutrone A, Lim HR, Kwon S, Choi JJ, Kim HS, Jang YC, Wolpaw JR, Yeo WH. Breathable, large-area epidermal electronic systems for recording electromyographic activity during operant conditioning of H-reflex. Biosens Bioelectron. 2020;165: Article 112404. 32729524 10.1016/j.bios.2020.112404 PMC7484316

[138] Thygesen K, Alpert JS, Jaffe AS, Chaitman BR, Bax JJ, Morrow DA, White HD, Executive Group on behalf of the Joint European Society of Cardiology(ESC)/American College of Cardiology (ACC)/American Heart Association(AHA)/World Heart Federation (WHF) Task Force for the Universal Definition of Myocardial Infarction. Fourth universal definition of myocardial infarction (2018). Circulation. 2018;138(20):e618–e651. 30571511 10.1161/CIR.0000000000000617

[139] Tian L, Zimmerman B, Akhtar A, Yu KJ, Moore M, Wu J, Larsen RJ, Lee JW, Li J, Liu Y, et al. Large-area MRI-compatible epidermal electronic interfaces for prosthetic control and cognitive monitoring. Nat Biomed Eng. 2019;3(3):194–205. 30948811 10.1038/s41551-019-0347-x

[140] Yang X, Wang S, Liu M, Li L, Zhao Y, Wang Y, Bai Y, Lu Q, Xiong Z, Feng S, et al. All-nanofiber-based janus epidermal electrode with directional sweat permeability for artifact-free biopotential monitoring. Small. 2022;18(12): Article e2106477. 35092161 10.1002/smll.202106477

[141] Wang C, Qi B, Lin M, Zhang Z, Makihata M, Liu B, Zhou S, Huang YH, Hu H, Gu Y, et al. Continuous monitoring of deep-tissue haemodynamics with stretchable ultrasonic phased arrays. Nat Biomed Eng. 2021;5(7):749–758. 34272524 10.1038/s41551-021-00763-4

[142] Hu H, Ma Y, Gao X, Song D, Li M, Huang H, Qian X, Wu R, Shi K, Ding H, et al. Stretchable ultrasonic arrays for the three-dimensional mapping of the modulus of deep tissue. Nat Biomed Eng. 2023;7(10):1321–1334. 37127710 10.1038/s41551-023-01038-w

[143] He W, Wang C, Wang H, Jian M, Lu W, Liang X, Zhang X, Yang F, Zhang Y. Integrated textile sensor patch for real-time and multiplex sweat analysis. Sci Adv. 2019;5(11):eaax0649. 31723600 10.1126/sciadv.aax0649 PMC6839936

[144] Sempionatto JR, Lin M, Yin L, De Lapaz E, Pei K, Sonsa-ard T, de Loyola Silva AN, Khorshed AA, Zhang F, Tostado N, et al. An epidermal patch for the simultaneous monitoring of haemodynamic and metabolic biomarkers*.* Nat Biomed Eng. 2021;5(7):737–748. 33589782 10.1038/s41551-021-00685-1

[145] Liu Z, Hu X, Bo R, Yang Y, Cheng X, Pang W, Liu Q, Wang Y, Wang S, Xu S, et al. A three-dimensionally architected electronic skin mimicking human mechanosensation. Science. 2024;384(6699):987–994. 38815009 10.1126/science.adk5556

[146] Matsuhisa N, Niu S, O’Neill SJK, Kang J, Ochiai Y, Katsumata T, Wu HC, Ashizawa M, Wang GJN, Zhong D, et al. High-frequency and intrinsically stretchable polymer diodes. Nature. 2021;600(7888):246–252. 34880427 10.1038/s41586-021-04053-6

[147] Wang W, Jiang Y, Zhong D, Zhang Z, Choudhury S, Lai JC, Gong H, Niu S, Yan X, Zheng Y, et al. Neuromorphic sensorimotor loop embodied by monolithically integrated, low-voltage, soft e-skin. Science. 2023;380(6646):735–742. 37200416 10.1126/science.ade0086

[148] Tilve-Martinez D, Neri W, Horaud D, Vukadinovic N, Berton B, Desmedt A, Yuan J, Poulin P. Graphene oxide based transparent resins for accurate 3D printing of conductive materials. Adv Funct Mater. 2023;33(21):2214954.

[149] Zeng C, Faaborg MW, Sherif A, Falk MJ, Hajian R, Xiao M, Hartig K, Bar-Sinai Y, Brenner MP, Manoharan VN. 3D-printed machines that manipulate microscopic objects using capillary forces. Nature. 2022;611(7934):68–73. 36289343 10.1038/s41586-022-05234-7

[150] Chen X, Jian W, Wang Z, Ai J, Kang Y, Sun P, Wang Z, Ma Y, Wang H, Chen Y, et al. Wrap-like transfer printing for three-dimensional curvy electronics. Sci Adv. 2023;9(30):eadi0357. 37494444 10.1126/sciadv.adi0357 PMC10371014

[151] Xue Z, Jin T, Xu S, Bai K, He Q, Zhang F, Cheng X, Ji Z, Pang W, Shen Z, et al. Assembly of complex 3D structures and electronics on curved surfaces. Sci Adv. 2022;8(32):eabm6922. 35947653 10.1126/sciadv.abm6922 PMC9365271

[152] Zhang W, Zhang L, Liao Y, Cheng H. Conformal manufacturing of soft deformable sensors on the curved surface. Int J Extreme Manuf. 2021;3(4): Article 042001.

[153] Yin J, Wang S, Tat T, Chen J. Motion artefact management for soft bioelectronics. Nat Rev Bioeng. 2024; 10.1038/s44222-024-00175-4. 10.1038/s44222-024-00175-4 PMC13012649 41884090

